# An Algal Nutrient‐Replete, Optimized Medium for Fast Growth and High Triacylglycerol Accumulation

**DOI:** 10.1002/pld3.70106

**Published:** 2025-09-12

**Authors:** Tim L. Jeffers, Ryan McCombs, Stefan Schmollinger, Srikanth Tirumani, Shivani Upadhyaya, Sabeeha S. Merchant, Krishna K. Niyogi, Melissa S. Roth

**Affiliations:** ^1^ Department of Plant and Microbial Biology University of California Berkeley California USA; ^2^ Quantitative Biosciences Institute University of California Berkeley California USA; ^3^ Department of Molecular & Cell Biology University of California Berkeley California USA; ^4^ Environmental Genomics and Systems Biology Division Lawrence Berkeley National Laboratory Berkeley California USA; ^5^ Howard Hughes Medical Institute University of California, Berkeley Berkeley California USA; ^6^ Molecular Biophysics and Integrated Bioimaging Division Lawrence Berkeley National Laboratory Berkeley California USA

## Abstract

Microalgae are promising sources to sustainably meet the global needs for energy and products. Algae grow under different trophic conditions, where nutritional status regulates biosynthetic pathways, energy production, and growth. The green alga *Chromochloris zofingiensis* has strong economic potential because it co‐produces biofuel precursors and the high‐value antioxidant astaxanthin while accumulating biomass when grown mixotrophically. As an emerging reference alga for photosynthesis, metabolism, and bioproduction, *C. zofingiensis* needs a defined, optimized medium to standardize experiments during fast growth for batch cultivation. Because the interplay of glucose treatment (+Glc) and mineral deficiency influences photosynthesis, growth, and the production of lipids and astaxanthin, we designed a replete nutrient medium tailored to the *C. zofingiensis* cellular ionome. We combined inductively coupled plasma mass spectrometry (ICP‐MS) and +Glc growth curves to determine a medium that is nutrient replete for at least 5 days of +Glc logarithmic growth. We found that there are high nutritional needs for phosphorus and sulfur during mixotrophy. Iron was the only element measured for which the cellular concentration correlated with exogenous concentration and was iteratively adjusted until the cellular ionome was consistent through the logarithmic growth phase. This *Chromochloris*‐Optimized Ratio of Elements (CORE) medium supports fast growth and high biomass and lipid accumulation without causing excess nutrient toxicity. This defined, nutrient‐replete standard is important for future *C. zofingiensis* investigations and can be adapted for other species to support high biomass for batch cultivation. The method used to develop CORE medium shows how ionomics informs replicable media design and may be applied in industrial settings to inform cost‐effective biofuel production.

## 1 Introduction

As global demands for energy and products rise, microalgae are gaining interest as components of a sustainable bioeconomy. Several algae species accumulate high quantities of lipids, making them relatively carbon‐neutral sources to replace fossil fuels (Unkefer et al. 2017; Arora et al. 2018). While many algae accumulate the biofuel precursors triacylglycerols (TAGs) under stress conditions at the expense of biomass (Wijffels and Barbosa 2010; Ma et al. 2022), some algae can amass high amounts of TAGs and biomass concurrently (Roth, Gallaher, et al. 2019; Jeffers et al. 2024). These algae are often mixotrophic, capable of both photosynthesis and organic carbon consumption for energy and biomass production (Suzuki et al. 2018; Blaby‐Haas and Merchant 2019). Taking advantage of algal mixotrophy can benefit the industrial cultivation of algae to produce lipids. For example, treating large‐scale cultures with reduced carbon and mineral nutrients via wastewater can reduce production costs (Ma et al. 2022). Furthermore, microalgae that produce high‐value compounds such as pharmaceuticals, nutraceuticals, and food supplements can improve the economic viability of biofuel through co‐production of valuable products. However, a growth medium that can remain nutrient replete to maximize biomass and biofuel and bioproduct accumulation while minimizing nutrient deficiency is necessary for industry and research.

The unicellular green alga *Chromochloris zofingiensis* (Chlorophyceae) is an emerging reference alga for research on photosynthesis, metabolism, and bioproduction (Roth et al. 2017; Roth, Gallaher, et al. 2019; Zhang et al. 2021; Wood et al. 2024). Not only is *C. zofingiensis* one of the highest algal producers of TAGs (Breuer et al. 2012), but it also co‐produces the high‐value nutraceutical astaxanthin (Roth et al. 2017; Jeffers and Roth 2021; Zhang et al. 2021). *C. zofingiensis* grows faster than the current industrial producer of astaxanthin, *Haematococcus lacustris* , and intriguingly, the organisms differ in their astaxanthin accumulation regulatory strategies (Jeffers and Roth 2021; Marcolungo et al. 2024). Furthermore, *C. zofingiensis* has a high‐quality complete genome that serves as a foundation for omics studies and accelerates bioproduct pathway discovery (Roth et al. 2017). Supplying sugars to *C. zofingiensis* cultures supports the accumulation of lipids, astaxanthin, and biomass concurrently (Roth, Gallaher, et al. 2019; Jeffers et al. 2024). Lipid accumulation during enhanced growth contrasts with the more widely studied phenomenon where nutrient deficiency induces storage lipids at the expense of biomass (Arora et al. 2018; Ma et al. 2022). A study comparing 96 species found that *C. zofingiensis* and *Tetradesmus obliquus*, both Sphaeropleales, had the highest TAG productivity under nitrogen starvation, a common condition used to induce TAG accumulation (Breuer et al. 2012). *C. zofingiensis* has evolutionarily distinct mechanisms of photosynthetic regulation and lipid accumulation compared to the reference green alga *Chlamydomonas reinhardtii* , possibly in part due to its ability to consume a wider array of carbon sources (Sun et al. 2008; Roth et al. 2017; Suzuki et al. 2018; Jeffers et al. 2024).

*C. zofingiensis* , like many algae, consumes exogenous sugars such as glucose (Glc) (Suzuki et al. 2018; Roth, Gallaher, et al. 2019). Glucose addition (+Glc) switches off *C. zofingiensis* photosynthesis and induces lipid accumulation via de novo fatty acid synthesis by a hexokinase‐dependent pathway (Roth, Gallaher, et al. 2019; Roth, Westcott, et al. 2019; Jeffers et al. 2024). Recently, we discovered that oxygenic photosynthesis is rescued when a replete iron supplement is added alongside +Glc, yet lipids accumulated with +Glc regardless of iron status (Jeffers et al. 2024). In contrast, *C. reinhardtii* cannot consume Glc, although it can be grown with acetate (Salomé and Merchant 2019). In addition to *C. zofingiensis*, other Sphaeropleales such as *Monoraphidium neglectum* , *T. obliquus,* and *Raphidocelis subcapitata* consume glucose and are oleaginous (Suzuki et al. 2018). Sugar consumption is also documented by Trebouxiophyceae green algae including *Auxenochlorella protothecoides* , *Chlorella vulgaris* , and *Chlorella sorokiniana* , where +Glc increases lipid and biomass productivity (Miao and Wu 2006; Gao et al. 2014; Rosenberg et al. 2014). Based on the research and bioindustry importance of the phenomenon of algal Glc consumption, it is essential to develop standardized growth protocols to understand algal Glc physiology and response.

While nutritional status reshapes algal metabolism, it can be difficult to identify due to “invisible” signatures of nutrient deficiency. For example, *C. reinhardtii* high‐affinity iron transport systems are activated in the iron‐deficiency regime (1–3 μM Fe) without any obvious phenotypic changes in chlorophyll content or quantum efficiency of PSII (Merchant et al. 2006; Glaesener et al. 2013). Ionomics, the identification of the mineral concentration within cellular biomass that is usually measured by inductively coupled plasma mass spectrometry (ICP‐MS), can aid in identifying nutrient needs and uptake mechanisms of an organism (Salt et al. 2008). Ionomics informed the determination of replete metal nutrition in *C. reinhardtii* through an experimental pipeline where a micronutrient supplement composition was derived from the cellular ionome of *C. reinhardtii* cultures measured through ICP‐MS (Kropat et al. 2011). The final supplement is threefold higher than the nutrient content of cellular biomass at stationary phase (Kropat et al. 2011), ensuring cells remained nutritionally replete with only a slight excess of mineral nutrients above their maximum biomass needs. At these concentrations, the ionome is consistent through logarithmic growth phase, indicating neither mineral nutrient deficiency nor hyperaccumulation due to excess media concentrations (Kropat et al. 2011; Hui et al. 2022). Ionomics data alongside careful understanding of cell physiology and nutrient treatment controls allow researchers to determine which cellular concentrations are optimal, scarce, or hyperaccumulated (Merchant et al. 2006; Long and Merchant 2008; Hui et al. 2022).

Regardless of the medium, adding Glc substantially improves biomass and TAG accumulation. Previous studies with *C. zofingiensis* have used “Proteose” medium, composed of Bristol's (Bold 1949), Chu's micronutrient supplement (Chu et al. 1975), and a Proteose peptone supplement (Roth et al. 2017; Roth, Gallaher, et al. 2019; Roth, Westcott, et al. 2019; Jeffers et al. 2024); modified Bristol's medium (Sun et al. 2008); and Kuhl medium (Huang et al. 2016; Zhang et al. 2017). As nutrient deficiency has unique metabolic impacts depending on +Glc vs. No Glc, it is imperative to design a standard *C. zofingiensis* medium to achieve a replete mineral composition as an experimental control for optimal +Glc growth and metabolism.

Here, we employed media design principles based on the ionome to ensure replete concentrations of mineral nutrients were optimized to the cellular ratio of elements in *C. zofingiensis* batch‐culture experiments. To account for the large increase in biomass in +Glc cultures of *C. zofingiensis* , we enhanced both macronutrient and micronutrient concentrations and adjusted for the trade‐off between excess nutrient toxicity in photoautotrophy and the high nutrient demands of +Glc cultures. Our final medium, *Chromochloris*‐Optimized Ratio of Elements (CORE), was both sufficient for high growth in +Glc yet maintained a consistent ionome during photoautotrophic logarithmic growth. This strategy shows how ionome measurements inform both replicable experimental design in the lab and mineral nutrient budgeting to enhance lipid production in algal bioprospecting systems, facilitating cost‐competitive alternatives to fossil fuels.

## 2 Results

### 2.1 Glucose Drives Fast Growth and High Biomass Accumulation, but Demands More Mineral Nutrients

Because +Glc induces high TAG and biomass accumulation in *C. zofingiensis* (Roth, Gallaher, et al. 2019; Roth, Westcott, et al. 2019; Jeffers et al. 2024), we designed a defined, minimal medium that compensated for the increased nutrient budget of +Glc batch cultures to keep cells nutrient replete regardless of trophic state. Because the Proteose medium provided an additional, undefined source of micronutrients and N‐rich organic compounds, we first redesigned the medium without the Proteose supplement and increased the micronutrient concentrations from Chu's supplement (Table S1), using similar stock sources and concentrations as optimized for *C. reinhardtii* (Kropat et al. 2011). Next, we increased macronutrients based on the physiological response in +Glc and preliminary spent medium measurements. For example, in replete Fe medium, increasing NO_3_^−^ caused cultures to remain green after +Glc addition (Figure S1), while similar cultures with replete Fe and NO_3_^−^ concentrations near Bristol's medium levels (3 mM) turned orange or brown with +Glc (Roth, Westcott, et al. 2019; Jeffers et al. 2024). Compared to Bristol's, NO_3_^−^ was increased to 20 mM. In addition, preliminary ICP‐OES measurements on spent +Glc medium suggested that +Glc increases the sulfur and phosphorus consumption of cultures. Therefore, S and P concentrations were also increased. These early modifications to the growth medium generally increased macronutrient concentration between ~1–6 fold and micronutrient concentration 0.2–60 fold, and we labeled this medium ADJ (for “adjusted”) (Table S1). With these mineral nutrient increases, ADJ medium became the base medium that was appropriate to fine‐tune *C. zofingiensis* medium components according to its general ionome needs and biomass levels in response to +Glc.

**FIGURE 1:**
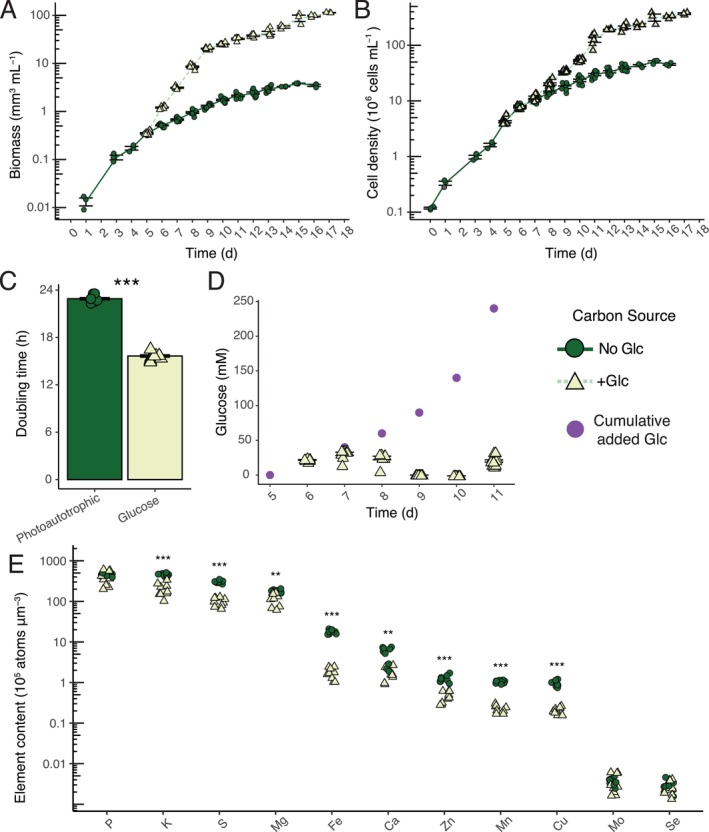
Glucose‐fed *C. zofingiensis* has fast, long‐term growth accumulating significant biomass. (A) Volumetric biomass and (B) cell density growth curves of batch cultures of control No Glc (dark green, circles, solid lines) and +Glc cultures (pale green, triangles, dotted line) in ADJ medium (Table S1). (C) Volumetric biomass doubling time (h) of No Glc log phase (calculated between days 1–6) growth and the first +Glc stage of log phase growth (days 5–8). (D) Glucose consumption during glucose resupply of batch experiments. The calculated addition of leftover glucose and added glucose is shown in purple. (E) Elemental content measured by ICP‐MS presented as atoms per volumetric biomass of No Glc vs. +Glc cells. Asterisks indicate level of significance of Welch's two‐tailed *t*‐test of the difference between +Glc vs. No Glc cells (* *p* < 0.05, ** *p* < 0.01, *** *p* < 0.001). Both the 10th day and 14th day ICP‐MS samples were combined for *t*‐test analysis and plotting. Values are expressed as means ± SE (*n* = 3–11).

The media design principles of Kropat et al. (2011) use maximum biomass (i.e., stationary phase) and the cellular ionome to calculate the required mineral nutrient composition for a replete growth medium. To determine a theoretical biomass maximum of +Glc resupply experiments, we aimed to see how high *C. zofingiensis* biomass becomes when Glc is not limited in batch cultures. To do so, 20 mM Glc was first added daily to logarithmic growth phase cultures (day 5, Figure 1) and Glc concentration in spent medium was measured daily to determine when Glc resupply needed to be increased to match consumption. +Glc immediately increased the logarithmic growth rate of mean culture volumetric biomass with a 15.65 ± 0.15 h doubling time between days 5 and 11 compared to the No Glc control doubling time of 22.91 ± 0.14 h (*t* (df) = 15.9, *p* = 1.67 × 10^−16^). Glc resupply was 60 mM day^−1^ by day 11 (Figure 1A–D). +Glc cells increased cell size, reaching a mean diameter of 9.4 ± 0.07 μm, which contrasts with control No Glc cells that had a mean diameter of 4.8 ± 0.04 μm. Cell density did not initially deviate from No Glc control until after 9 days when +Glc cell density did start exceeding No Glc (Figure 1B). To incorporate the cell size data, we report the cumulative volumetric sum (mm^3^) per milliliter of cells as a more accurate description of biomass than the more commonly used cell density, which is consistent with previous publications (Roth, Gallaher, et al. 2019; Roth, Westcott, et al. 2019; Jeffers et al. 2024). We provide cell density information in Supporting Information.

Due to continued growth and high Glc consumption, a second experimental phase involved resuspending the cultures in fresh medium with 500 mM Glc every 2–3 days. The experiment continued until day 24, when cellular volume was approximated as ~22.5% of total liquid culture volume (Figure S2B). The large increase in biomass produced when Glc is not limiting showed a promising industrial possibility of growing high‐density algal slurries with lower water volume needs. However, for laboratory studies trying to achieve these maximum biomass levels may create unrealistically high nutrient compositions.

### 2.2 Identifying the Cellular Ratio of Elements of *C. zofingiensis* Cells

The composition of elements of cells can be multiplied by a target biomass to calculate a replete nutrient budget (Kropat et al. 2011). To measure the ionome, cellular concentrations of P, K, S, Mg, Ca, Fe, Mn, Zn, Cu, Se, and Mo were determined by ICP‐MS in No Glc and +Glc cultures on days 10 and 14 of the *C. zofingiensis* growth curve (Figure 1E). Most mineral nutrients displayed significantly lower concentrations per biomass in +Glc vs. No Glc at both time points (*p* < 0.05, full statistical reporting in Table S2) except for P, Mo, and Se, for which +Glc vs. No Glc concentrations were not significantly different. We suspect the decrease of mineral nutrients per volumetric biomass is due to carbon‐dense starch and lipid accumulation dominating cellular space in +Glc (Roth, Gallaher, et al. 2019; Jeffers et al. 2024).

*C. zofingiensis* had lower Ca requirements relative to other photosynthetic organisms. While Ca is defined as a macronutrient and Fe as a micronutrient, we observed that Ca levels were lower than Fe levels in *C. zofingiensis* No Glc cells (respectively, 0.52 ± 0.075 × 10^6^ Ca atoms μm^−3^ and 1.79 ± 0.068 × 10^6^ Fe atoms μm^−3^) (Figure 1E). In contrast, in *C. reinhardtii* Ca is more abundant than Fe in log‐phase growth and conditions where iron is not in excess (Merchant et al. 2020; Schmollinger et al. 2021; Hui et al. 2022), and Ca can compose 0.1%–5% of vascular plants' dry weight (Thor 2019).

### 2.3 Optimizing Nutrient Ratios Improves Glucose‐Fed Growth

**FIGURE 2:**
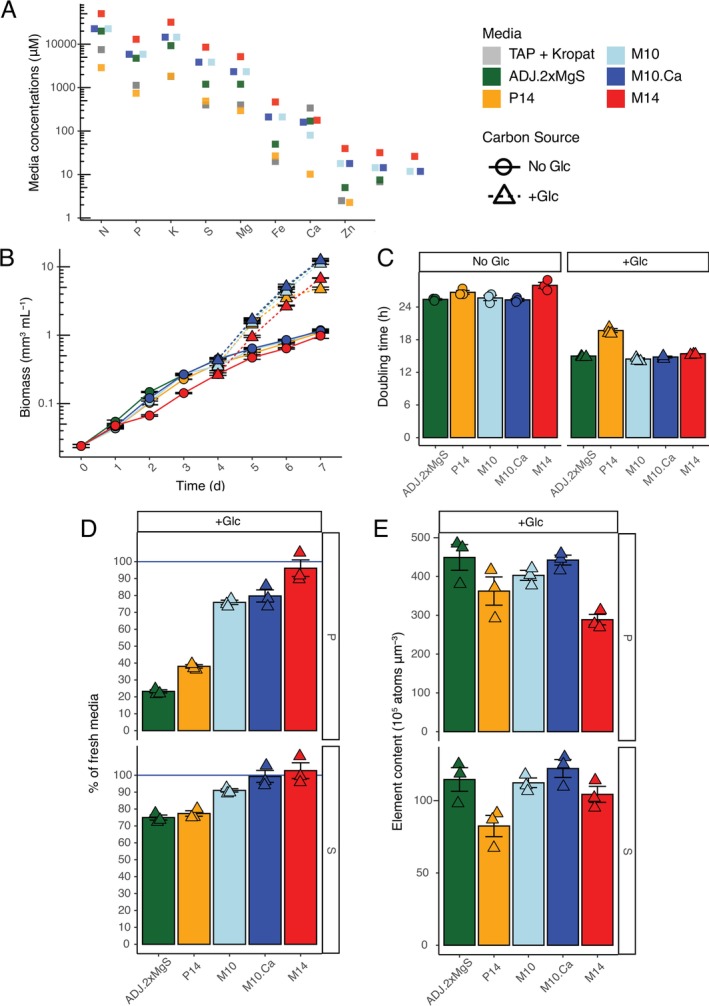
Nutrient concentration trade‐offs for No Glc and long‐term +Glc growth. (A) Elemental nutrient concentrations of media designed to meet nutrient demands of different stages of growth: No Glc (P14, orange) and long‐term +Glc growth (M10, light blue; M10.Ca, blue; M14, red) (Table S1). Comparison media for reference are the previously described ADJ medium (green) and micronutrient‐optimized TAP medium for *C. reinhardtii* (Kropat et al. 2011, gray). (B) Volumetric biomass growth curve of *C. zofingiensis* in media ADJ, P14, M10, M10.Ca, and M14 for No Glc (solid line, circles) and +Glc treatment (dotted line, triangles). Each point is mean values instead of raw due to high overlap in growth rates. Figure S3A contains corresponding cell density data. (C) Volumetric biomass doubling time (h) across media and trophic states. Points are individual growth curves. (D) Percentage of sulfur (S) and phosphorus (P) left in spent medium relative to fresh medium in the +Glc condition on day 7 (3 days of +Glc growth). Concentrations are measured by ICP‐MS. (E) Cellular elemental content of S and P presented as atoms per volumetric biomass in day 7 + Glc cells. Values are expressed as means ± SE (*n* = 3).

We tested the trade‐off between nutrient excess toxicity vs. insufficiency by creating scalar multipliers of the ionome and observing the impacts on algal growth. New media recipes were derived by multiplying the mean elemental concentration per biomass in photoautotrophy by the biomass in the following growth stages: photoautotrophic (No Glc) stationary stage at day 14 (called P14, the lowest nutrient composition), mixotrophic (+Glc) at day 10 (M10), and mixotrophic (+Glc) at day 14 (M14, highest nutrient composition) (Figure 2A, Table S1 for media recipes). For M10, we additionally designed a medium (M10.Ca) that had two‐fold higher Ca concentration to ensure that the previously measured low Ca (Figure 2A) was not a result of a nutrient deficiency in ADJ medium. Like the media optimization for *C. reinhardtii* , this composition was multiplied by three for mild nutrient excess and to maintain a similar nutrient environment during logarithmic growth (Kropat et al. 2011). However, unlike for *C. reinhardtii* , macronutrient levels also required adjustments to compensate for +Glc biomass increases. Standard ICP‐MS protocols use nitric acid for digestion of cell material, preventing the accurate determination of the cellular N content. The NO_3_^−^ concentration was therefore controlled to be at the same ratio to all other elements across tested media. In +Glc‐derived M10 and M14 medium, all macronutrients and micronutrients (except Ca) exceeded the composition of revised *C. reinhardtii* TAP medium with micronutrient supplement (Kropat et al. 2011) and the originally used ADJ medium, suggesting that +Glc biomass levels greatly enhance nutrient budget demands (Figure 2A).

No Glc and +Glc growth curves across media revealed a trade‐off between nutrient limitation and excess nutrient toxicity (Figure 2). A one‐way ANOVA of No Glc biomass doubling time showed significant variation due to medium type (*F* (4, 10) = 8.7, *p* = 2.7 × 10^−3^). The highest nutrient medium M14 had the slowest No Glc biomass doubling time (28.0 ± 0.55 h vs. 25.7 ± 0.49 h for M10, Figure 2B,C, *p‐*adj = 0.011), and suppressed cell division compared to all other media (indicated by change in cell density, Figure S3A). There was no significant No Glc doubling time variation between the M10, M10.Ca, ADJ, and P14 media (Figure 2B,C, *p‐*adj > 0.05). Because M14 had the lowest rate of No Glc growth, one or more elements may have induced nutrient toxicity physiology in No Glc cultures. +Glc doubling times were also significantly impacted by medium type (*F* (4, 10) = 118.27, *p* = 2.2 × 10^−8^). In the lowest nutrient medium P14, growth rate declined by the second day of +Glc treatment, with a doubling time of 19.7 h ± 0.35 h (vs. M10: 14.4 ± 0.21 h, *p‐*adj = 3.6 × 10^−8^) confirming P14 was insufficient for maximum +Glc growth (Figure 2C). Altogether, these results suggest that the macronutrient concentrations of M10 were optimal for both No Glc and +Glc growth.

Doubling Ca in M10.Ca did not change growth (25.7 ± 0.49 h doubling time, No Glc) or the cellular Ca concentration compared to M10 (25.3 ± 0.24 h doubling time, No Glc), and thus M10 was determined to be sufficient for *C. zofingiensis* medium (Figure 2C). Interestingly, P14 cells accumulated ~3.7–4.5 fold more Mn and ~1.3–1.8 more Mg than all other media, which was confirmed by depletion of these mineral nutrients in the spent medium (Figure S3B,C). While growth was not significantly limited in No Glc P14 growth, the unique hyperaccumulation further suggested that P14 levels altered nutrient import physiology, potentially as a signaling response to low nutrient concentrations in P14.

ICP‐MS measurements of spent media indicated concentration adjustments were required to maintain replete nutrient levels through +Glc logarithmic growth phase. While ADJ and the M10 medium had similar growth, the spent medium of ADJ after +Glc treatment had more significant macronutrient losses. For example, ~73% of P and ~25% of S were depleted in ADJ spent medium after 3 days of +Glc, while M10 medium was reduced ~25% P and ~9% of S after 3 days +Glc (Figure 2D). These losses were not reflected by a unique hyperaccumulation of S or P per biomass in ADJ and indicate that the initial S and P increases from Bristol's to make ADJ were still not sufficiently replete (Figure 2E). The macronutrient supplement of M10 was thus determined as more appropriate than ADJ for maintaining +Glc cultures in a replete state. Since the M10 medium is designed to support, in threefold excess, the biomass of cultures achieved after 5 days of +Glc treatment, we anticipated cells with M10 levels of nutrients should remain physiologically replete of all measured mineral nutrients for ~5 days of continuous Glc consumption.

### 2.4 Optimizing pH, Nitrogen, Potassium, and Sodium for Replicability

After using ICP‐MS to determine replete nutrient concentrations to support +Glc growth in the design of the base medium M10, we further optimized other environmental variables for consistent growth experiments.

First, we evaluated ideal pH and buffers that would enhance growth and maintain environmental replicability of M10 medium. Throughout the optimization, we found that *C. zofingiensis* delayed biomass accumulation in mildly acidic versions of M10 medium. For example, mean volumetric biomass three days after inoculation of pH 6.0 and pH 6.5 cultures was, respectively, 4.9 ± 1.65 × 10^7^ μm^3^ mL^−1^ and 9.65 ± 1.94 × 10^7^ μm^3^ mL^−1^, whereas all cultures ≥ pH 7.0 had mean volumetric biomass 21.14 ± 0.59 × 10^7^ μm^3^ mL^−1^ (Figure S4A,B). An ANOVA revealed the significant effect of pH (*F* (6, 29) = 27.58, *p* = 9.8 × 10^−11^) and the post hoc Tukey's HSD test indicated a significant difference (*p*‐adj < 0.05) between pH 6.0 and pH 6.5 media with all media ≥ pH 7.0 in 8 of 11 pairwise comparisons but did not show a significant difference among all media ≥ pH 7.0. From these findings, initially, ADJ medium and M10 were adjusted to a pH of 8.0 with phosphate as buffer (Table S1). However, across three independent experimental replicates, we were unable to detect significant differences between pH 7.5 and pH 8.0 (*p‐*adj = 0.944) in No Glc growth curves. To reduce the increase in nutrient precipitation effect that would occur with excess alkalinity and to use a pH amenable to the pK_a_ of common laboratory buffers (e.g., HEPES), we lowered M10 medium pH to 7.5.

We then tested buffers suited for maintaining pH 7.5 to ensure they had no effect on algal physiology compared to a control medium that was only buffered by K_2_HPO_4_/KH_2_PO_4_, which is assimilated by cells (Figure 2D), reducing the buffer capacity of the media during growth. Growth curves of 20 mM HEPES and Tris buffers and the phosphate control showed no difference in volumetric biomass growth, indicated by an ANOVA on Day 7 cultures (*F*(2, 13) = 0.95, *p* = 0.41; Figure S4A). However, an ANOVA showed a significant impact of buffer type on cell density (*F* (2, 13) = 33.37, *p* = 7.6 × 10−^6^) and mean cell diameter (*F* (2, 13) = 18.19, *p* = 1.7 × 10^−4^). Tris‐treated cultures had significantly lower cell density on Day 7 (1.19 ± 0.03 × 10^7^ cells mL^−1^) and larger individual cells compared to both HEPES (1.65 ± 0.08 × 10^7^ cells mL^−1^, *p*‐adj *=* 2.6 × 10^−4^) and control cultures (1.84 ± 0.07 × 10^7^ cells mL^−1^, *p*‐adj = 7.5 × 10^−6^; Figure S4E,D). All growth parameters were not significantly different between HEPES and control (*p*‐adj = 0.104–0.456; Figure S4E,D). Measurements of the pH of spent media showed that phosphate buffer control of No Glc cultures increased alkalinity from pH 7.5 to pH > 8.5 over 7 days, but HEPES buffer could maintain pH at ~7.5 for 7 days of No Glc growth. With its strong pH control and no indication of impact on physiology, we chose HEPES buffer to improve M10 medium's (now called M10 HEPES, Table S1) environmental consistency through logarithmic growth.

We next assessed nitrogen levels and sources manually, as we could not detect this compound by ICP‐MS. Nitrogen is considered the most abundant biological element after carbon, oxygen, and hydrogen (Merchant et al. 2020). While algae would invest less energy in nitrate reduction if they were provided ammonium (NH_4_^+^), we found that *C. zofingiensis* did not grow in concentrations ≥ 22.5 mM NH_4_Cl (Figure S5A,B). *C. zofingiensis* could also grow in 22.5 and 45 mM NaNO_3_ with no apparent growth defects. To ensure NO_3_^−^ levels could be both high and proportionally greater than any other nutrient in the medium, we increased M10 medium levels from 22.5 to 45 mM NO_3_^−^ (now called M10 HEPES N Boost, Table S1) to keep nitrogen in excess for replete nutrient growth and avoid nitrogen deprivation in +Glc.

K and Na cellular concentrations could not be matched to the optimized ratio of elements of cells due to their co‐occurrence in the salts used to prepare the medium (e.g., K_n_H_n_PO_4,_ NaNO_3_). Several plant studies show that the ratio of K/Na ions is important in salinity responses (Schachtman and Liu 1999). The ratio of K/Na in this defined medium was adjusted by varying source salts containing phosphate and nitrate (e.g., KNO_3_ vs. NaNO_3_). To ensure cellular health, we compared a gradient of different K/Na ratios from 1:5 to 5:1 and found no significant impact of medium on growth rate (ANOVA: *F* (5, 15) = 2.26, *p* = 0.101; Figure S6). Rather than requiring additional steps to incorporate various K/Na salts, our medium recipes only use single sources of NaNO_3_ and K_n_H_n_PO_4_ salts.

### 2.5 Adjusting Iron for a Consistent Ionome in Logarithmic Growth

**FIGURE 3:**
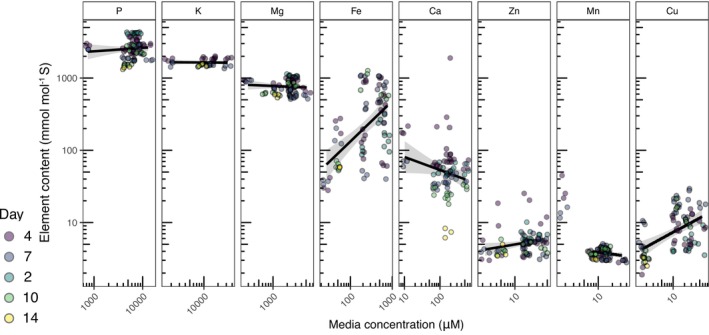
Nutrient levels across different external nutrient concentrations. Ionome variation per element normalized by cellular sulfur concentration across 81 No Glc samples under diverse media regimes and sampling time points (point color represents day after inoculation). The fresh medium concentration of each element per samples is represented on the *x*‐axis of each panel, whose range varies according to nutrient. The black line represents the linear regression conducted on cellular elemental concentration as a function of medium concentration and results of each element's linear model are found in Table S3. For Mn, 12 P14 biological samples were plotted but not considered in the linear regression as they represented a hyperaccumulation in low external nutrient concentration that was not reflected in the range of higher concentration (Figure S2). Gray regions represent 95% confidence intervals of the regression model (*n* = 81).

Finally, we adjusted the medium to ensure the ionome was consistent through the logarithmic growth phase, which is essential to reduce time‐dependent confounding effects in time‐course studies. A previous study on *C. zofingiensis* showed that an iron supplement (≥ 10 μM Fe) rescues photosynthesis and further increases the biomass of +Glc cultures (Jeffers et al. 2024), indicating that the undefined amount of iron in Proteose (estimated between 1 and 2 μM, M. Meagher pers. comms. 2024) was at a limiting concentration. As hyperaccumulation can be a feature of nutrient excess (Hui et al. 2022), we evaluated the relationship between external media concentration and the ionome levels of No Glc biomass across the media variations and timepoints we obtained through ICP‐MS data (Figure 3). Nutrient concentrations were normalized to moles of S, a method to standardize comparisons across different experiments (Schmollinger et al. 2021; Hui et al. 2022). Promisingly, most elements had consistent concentration per S despite ≥ 10‐fold external range concentration of each element, showing external concentration usually does not impact nutrient import (Figure 3, Table S3). However, iron was the largest outlier, showing increasing cellular concentration and variability as its external concentration exceeded 100 μM. A linear model of external to internal concentration indicated a positive slope of 0.501 mmol (mol S)^−1^ increase in cellular Fe per μM increase in external Fe (*p* = 7.34 × 10^−5^). To a lesser extent, cellular copper also increased with external concentration (*p* = 0.01), but the mean increase plateaued above 10 μM external Cu, suggesting Cu quotas were saturated.

We compared iron quotas across *C. reinhardtii* and *C. zofingiensis* to determine that this nutrient responsive pattern was not due to *C. zofingiensis* simply requiring very high iron quotas. The M10 medium predicted a need for ~210 μM Fe for biomass growth, and the determined replete iron quota is 20 μM Fe for *C. reinhardtii* (Kropat et al. 2011). Furthermore, at similar concentrations to M10 medium (200 μM), *C. reinhardtii* hyperaccumulates iron in an excess state with no obvious impact on physiology or growth in standard conditions (Long and Merchant 2008; Hui et al. 2022). Therefore, we suspected the media concentration dependence of cellular iron accumulation was due to a conserved green algal excess iron response and not due to specifically high iron needs in *C. zofingiensis* .

**FIGURE 4:**
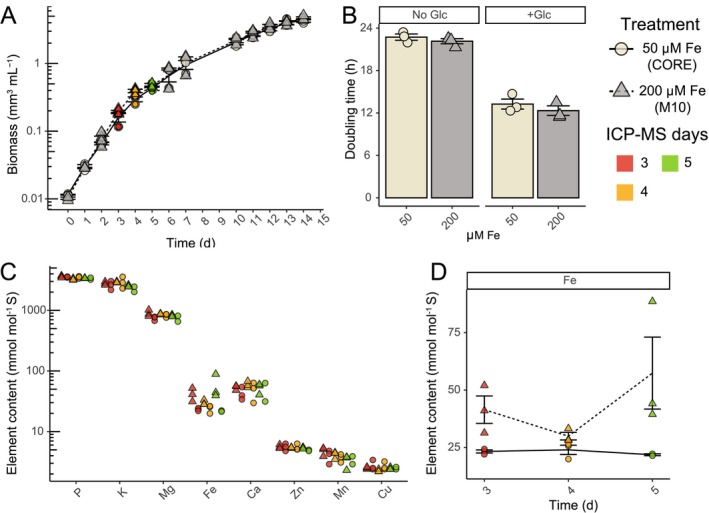
A consistent ionome is maintained through No Glc logarithmic growth. (A) Volumetric biomass growth curve of M10 HEPES N Boost medium, No Glc with 200 μM Fe (gray, triangle, dotted line) vs. 50 μM Fe (beige, circles, solid line). Days 3, 4, and 5 samples were collected for ionomic quantification and are overlayed with red, orange, and green, respectively*.* (B) Volumetric biomass doubling time (h) of 50 vs. 200 μM Fe treatments in No Glc and +Glc (140 mM Glc, single dose). Figure S7 contains corresponding cell density data. (C) The elemental content (normalized by cellular sulfur concentration) of all measured elements through log phase between 50 (circles) vs. 200 (triangles) μM Fe across days 3, 4, and 5. (D) Close‐up of cellular iron concentration across 50 vs. 200 μM Fe across days 3, 4, and 5. Because of this result, the 50 μM Fe medium represents the finalized CORE medium from this study. Values are expressed as means ± SE (*n* = 2–3).

We tested if Fe concentration could be lowered to create a consistent ionome at the logarithmic growth phase. Cells were grown in M10 medium with 50 or 200 μM Fe, and all other nutrient concentrations remained constant. No significant growth change in biomass (Figure 4A,B) or cell density (Figure S7) occurred between iron treatments through No Glc growth or after +Glc treatment. The ionome variation was sampled throughout the No Glc log phase (days 3–5). All elements including Fe had consistent logarithmic growth phase ionomes at 50 μM Fe (Figure 4C). However, Fe was the only element with log phase accumulation differences at 200 μM Fe (Figure 4), where it was higher and had greater variation at 200 μM Fe compared to 50 μM Fe (Figure 4D). To create a consistent ionome, we adjusted the final medium to 50 μM Fe, avoiding both deficiency and excess nutrient impacts of *C. zofingiensis* during No Glc and +Glc logarithmic growth. Based on using ionomics to define the optimized ratio of mineral nutrients for replete growth, we named the final growth medium *C. zofingiensis* Optimized Ratio of Elements or CORE medium.

The final recipe for CORE medium can be found in Appendix S1 and can be used for controlled experiments comparing +Glc and No Glc physiology as well as a reference point to derive nutrient deficiency parameters and could be applicable to other microalgae particularly under mixotrophic or heterotrophic growth.

### 2.6 CORE Medium Improves Biomass and Lipid Productivity

**FIGURE 5:**
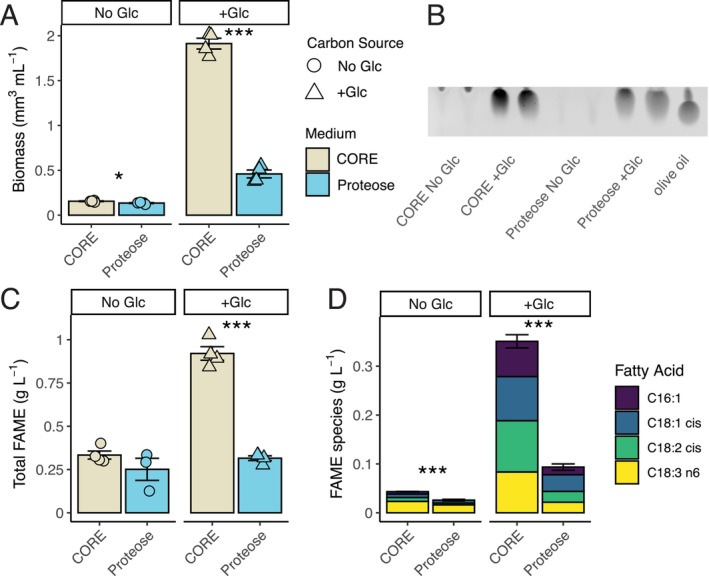
CORE enhances growth and lipid productivity of +Glc cultures. (A) Volumetric biomass of CORE (beige) and Proteose (light blue) cultures in +Glc (triangle) and control No Glc (circles), 96 h after +Glc treatment. Figure S8A contains corresponding cell density data. (B) TLC showing high TAG accumulation at 96 h + Glc (equal culture volume loaded for samples, 2 replicates each) with 1 μL of olive oil used as a TAG standard. (C) Total lipid productivity of cultures (g L^−1^) based on the sum of all identified FAME peaks quantified by GC‐FID. (D) Lipid productivity of a subset of the FAMEs of fatty acid species that are known to be enriched in TAG. Asterisks indicate level of significance of Welch's two‐tailed *t*‐test of the difference between +CORE vs. Proteose cultures (* *p* < 0.05, ** *p* < 0.01, *** *p* < 0.001). Values are expressed as means ± SE (*n* = 3–4). For (D), SE is of the total represented FAMEs.

Having found CORE medium improves experimental replicability and growth to sustain +Glc‐fed biomass, we then tested if the CORE medium improved biomass and total lipid productivity compared to Proteose medium. We conducted an experiment growing *C. zofingiensis* in both CORE and Proteose media to compare impacts of +Glc treatment (Figure 5). After 96 h, CORE+Glc had ~4.2 fold higher biomass than Proteose+Glc (*t* (df) = 5.5, *p* = 2.8 x 10^−4^; Figure 5A, Figure S8), confirming the medium optimization enhanced +Glc growth. In No Glc conditions, CORE biomass was also moderately yet significantly increased from Proteose (respectively, 1.3 ± 0.04 x 10^8^ μm^3^ mL^−1^ and 1.6 ± 0.04 × 10^8^ μm^3^ mL^−1^, *t* (df) = 5.7, *p* = 0.019). The improvement from CORE and Glc resulted in a ~14.2 fold higher biomass in CORE+Glc than Proteose No Glc (*t* (df) = 3.04, *p* = 7.8 × 10^−5^).

We also found that CORE+Glc had more TAG and lipid accumulation than Proteose+Glc. Thin Layer Chromatography (TLC) of the lipid extracts of 96 h cultures showed high TAG accumulation in CORE+Glc and Proteose+Glc, and TAG was not detected in No Glc samples of either medium (Figure 5B). Since the intensity of the CORE+Glc bands was more saturated than the Proteose+Glc bands in TLC, we measured total lipid bioproduction through fatty acid methyl esters (FAMEs) species that were identified through gas chromatograph with flame ionization detection (GC‐FID). After 96 h, total FAME of cultures of CORE+Glc was ~2.9 fold higher than Proteose+Glc (respectively, 0.92 ± 0.04 g L^−1^ and 0.32 ± 0.01 g L^−1^; Figure 5C; *t* (df) = 3.71, *p* = 2.1 × 10^−4^). The sum for FAMEs of C16:1, C18:1 *cis*, C18:2 *cis*, and C18:3 *n6*, which have been previously found in *C. zofingiensis* to be enriched in TAG molecules (Wu et al. 2021), indicated TAG accumulation was ~3.8 fold higher in CORE+Glc than Proteose+Glc (respectively, 0.35 ± 0.013 g L^−1^ and 0.09 ± 0.006 g L^−1^; *t* (df) = 4.33, *p* = 3.8 × 10^−5^; Figure 5C). Additionally, these FAMEs were higher in both +Glc cultures compared to their No Glc medium controls (Figure 5C; Proteose *t* (df) = 3.43, *p* = 1.2 × 10^−3^, CORE *t* (df) = 3.01, *p* = 1.8 × 10^−4^). Furthermore, CORE+Glc had ~13.6 fold higher sum of TAG‐associated FAMEs than Proteose No Glc (*t* (df) = 3.1, *p* = 1.23 × 10^−4^). In total, the optimized CORE medium showed increases to biomass, total lipid productivity, and storage lipid productivity in +Glc conditions.

Lastly, we compared the production of ketocarotenoids between the two media. High Performance Liquid Chromatography (HPLC) revealed high production of astaxanthin under +Glc treatments of both media that were low or negligible in No Glc controls (Figure S9). The astaxanthin peaks were similar in area between the two media despite CORE+Glc cultures appearing green and Proteose+Glc cultures appearing orange (Figure S9A). Peaks associated with chlorophyll *a* and chlorophyll *b* were much higher in CORE+Glc than in Proteose+Glc, explaining why CORE+Glc cells remain green while still producing astaxanthin (Figure S9B). While it is likely that additional stresses like low nitrogen or high light could further increase astaxanthin production, +Glc induces astaxanthin production even in the high nutrient optimum states supported by CORE medium.

## 3 Discussion

CORE medium was designed based on the cellular ionome of *C. zofingiensis* to budget for +Glc growth while avoiding nutrient toxicity and maintaining replicability of log‐phase cultures during batch cultivation. Balancing the trade‐off of limitation vs. toxicity makes CORE a suitable reference to study how nutrient deficiency acts in No Glc vs. +Glc cultures. Experiments aiming to understand long‐term +Glc cultures, beyond the 5‐day window supported by CORE, may require chemostats or turbidostats as more appropriately controlled systems. However, as it is unfeasible to have the number of chemostats needed for experiments that require a high sample number (such as combinatorial nutrient time courses in Jeffers et al. 2024), CORE medium is appropriate for multiomic investigations of nutritional status and their interplay. CORE medium may also be useful for other microalgae species that achieve high biomass, particularly through mixotrophic or heterotrophic growth. We have successfully grown another oleaginous alga, *Auxenochlorella* sp. UTEX 250‐A, in CORE medium with slight modifications based on species‐specific physiology, despite being evolutionarily distant from *C. zofingiensis* . Altogether, having a defined, nutrient‐replete medium to support high biomass and bioproducts is essential for understanding these green algae.

Large macronutrient (N, P, K, Mg, and S) supplements to the original Bristol's medium were required to maintain replete concentrations in +Glc growth. Analysis of spent medium from +Glc cultures showed that S and P were highly consumed, indicating these elements are in specific demand in +Glc growth. Our previous research indicates Glc induces sulfur‐related biosynthetic pathways (METE, methionine synthase; THIC and THI1/4 for thiazole biosynthesis) in *C. zofingiensis* , particularly during a switch‐off of photosynthesis (Jeffers et al. 2024). +Glc also upregulates de novo fatty acid biosynthesis, which requires the S‐containing coenzyme A (Roth, Gallaher, et al. 2019; Jeffers et al. 2024). These highly expressed pathways could drive increased S uptake and demand in +Glc (Jeffers et al. 2024). Likewise, increases in the major P sink of carbohydrate phosphorylation and rRNA synthesis could underlie the higher P uptake in +Glc (Sulpice et al. 2014). As these S‐ and P‐linked metabolic processes are connected to lipid and carbohydrate metabolism and biomass, their connection to +Glc signaling should be considered for metabolic engineering and design of large‐scale bioprospecting of lipids.

Measuring the ionome of cells grown in nutrient compositions of a range of concentrations showed that across the tested conditions, the relative cellular compositions of most mineral nutrients are not influenced by increasing external concentrations. Fe is an exception, as its cellular concentration generally increased with external concentration. Although *C. reinhardtii* has different metabolic and bioproduction features compared to *C. zofingiensis, C. reinhardtii* 's ionome has been extensively studied (Merchant et al. 2020; Schmollinger et al. 2021; Hui et al. 2022) and is therefore a comprehensive reference for comparative ionomics. This pattern of *C. zofingiensis* excess iron accumulation is consistent with the *C. reinhardtii* iron accumulation (Long and Merchant 2008; Schmollinger et al. 2021; Hui et al. 2022), where both organisms show accumulation patterns at ~200 μM Fe. Like *C. zofingiensis* , iron hyperaccumulation in *C. reinhardtii* does not impact growth under standard conditions (Figure 4A,B; Long and Merchant 2008; Hui et al. 2022). However, *C. reinhardtii* cells with hyperaccumulated iron are sensitive to high‐light treatment compared to replete Fe‐treated cells (Long and Merchant 2008). Understanding accumulation patterns should be considered for media design across species. Beyond ensuring experimental replicability of the ionome (Hui et al. 2022), avoiding elemental accumulation in a medium will prevent confounding influences of non‐nutrient‐related studies. For example, researchers studying high light or reactive oxygen species response may want to ensure observed stress responses are not due to accumulated micronutrients exacerbating the impacts of the stress.

Furthermore, we noted in our study that it is important to detect the physiological impacts of buffer choice. Our study shows that an organic buffer supplement was necessary to maintain pH through log phase. Overall biomass was similar between Tris, HEPES, and cultures lacking organic buffers, indicating that the organic buffers did not impact carbon assimilation into biomass. While the cell density was reduced in Tris buffer, the cells were slightly larger in size compared to HEPES or cultures lacking organic buffers. Because of the effect on cell division in Tris, albeit with no cost to biomass, we chose to use HEPES buffer to have indistinguishable cell physiology compared to nonbuffered controls. While phosphorus components of growth medium are known for acting as both a nutrient and a buffer, higher phosphorus also increases the propensity of media to precipitate with divalent cations like Mg^2+^ and Ca^2+^. We also based the concentrations of mineral nutrients in CORE to have the same ratio as calculated from the ionome. We found the addition of HEPES buffer was necessary to maintain pH through logarithmic growth, as have previous publications (Zepernick et al. 2020).

Measuring spent media and cellular concentration of elements in algal biomass by ICP‐MS, as pioneered by Kropat et al. (2011), creates a framework to discover elemental demands and identify an ideal standardized nutrient composition. From this reference medium, all other nutrient regimes and physiologies can be determined. The considerations of this technique may be optimized in industrial contexts to improve production costs. Biofuel production is often hindered by the costs of nutrients and the biomass loss that occurs during most green algal lipid induction by nutrient deprivation (Zhang et al. 2021; Ma et al. 2022). For industrial‐scale biofuel production, measurements of spent media and ionome may be calculated with their impact on biofuel and carotenoid production. This strategy may improve the yield and cost efficiency of valuable bioproducts. Therefore, consideration of algal optimal nutrient needs could serve as a reference point to find metabolic pathways (bioengineering targets) and nutrient adjustments (economic targets) that improve the sustainability of algae as viable replacements for fossil fuels.

While media rich with mineral nutrients and carbon sources may produce high levels of biofuels and bioproducts, the mineral nutrient and exogenous carbon supplements can be costly. Fortunately, microalgae can capitalize on mineral nutrients and carbon sources from waste streams to reduce costs. The most promising source of carbon may be byproducts from table sugar manufacturing, which can be abundant with glucose, sucrose, and fructose (Liu et al. 2012). *C. zofingiensis* has been shown to accumulate biomass, lipids, and astaxanthin when grown with industrial waste from cane molasses (Liu et al. 2012). Furthermore, *C. zofingiensis* has been noted for its superior capabilities for both nitrogen and phosphorus uptake, showing that this alga can simultaneously treat wastewater while accumulating bioproducts (Zhao et al. 2018). Models have also shown competitive biodiesel prices using waste streams and a combination of microalgae ( *C. reinhardtii* ) and yeast (Gomez et al. 2016). With the understanding from this study of the nutrient profile that leads to optimal biomass, lipid, and astaxanthin accumulation, waste streams can be orchestrated for efficient and economically feasible bioproduction and wastewater treatment.

The CORE medium, designed based on the cellular ionome of mixotrophic cells, establishes a defined, nutrient‐replete standard for future studies focused on *C. zofingiensis* . This medium is optimized for fast growth and can support high biomass and production of TAGs in *C. zofingiensis* and can be used in mixotrophic and heterotrophic growth of other microalgae. In addition to batch liquid culture experiments described in this study, we have found this medium functions for long‐term maintenance of cells on agar plates. Additionally, this standardized medium allows for controlled comparisons between experiments and laboratories and could be useful in studies that investigate the interplay of energy metabolism with nutrient demands and cofactors. This study also provides a strategy to improve yield and economic feasibility of microalgae and a nutrient profile to match from waste streams to improve the cost efficiency of algal bioproducts.

## 4 Experimental Procedures

### 4.1 Media Concentrations

Expanding from the initial medium described in Roth, Gallaher, et al. (2019), *C. zofingiensis* SAG 211‐14 (Roth et al. 2017) cultures were grown in iterative heterotrophic (+Glc) media recipes that were altered based on improvements to biomass, photosynthesis, and chlorophyll production from the Proteose medium (Table S1). ADJ medium, the base medium for this study, was composed of adjustments to Bristol's macronutrient base (Bold 1949). Stock solutions of metal micronutrients were complexed to Na_2_‐EDTA, as described by Kropat et al. 2011 (see Appendix S1 for details). Like in *C. reinhardtii* (Kropat et al. 2011), we did not detect cellular concentrations of cobalt or boron in *C. zofingiensis* or find any impact of these elements on growth; therefore, we excluded these elements starting with ADJ medium. In Figure 1, ADJ with 0.6 mM MgSO_4_ and 1.2 mM MgSO_4_ was tested simultaneously with no significant growth differences and was combined in Welch's two‐tailed *t‐*test of No Glc vs. +Glc. Because sulfur had higher cellular concentrations than its bound cation Mg, an additional K_2_SO_4_ supplement was added to the medium.

The finalized CORE medium contains 45 mM NaNO_3,_ 2.5 mM MgSO_4_, 1.5 mM K_2_SO_4_, 0.08 mM CaCl_2_, 5.7 mM K_2_HPO_4_, and 0.3 mM KH_2_PO_4_ (Table S1, Appendix S1). Micronutrients included 50 μM Fe‐EDTA, 15 μM Mn‐EDTA, 12 μM Cu‐EDTA, and 17.5 μM Zn‐EDTA. Selenium and molybdenum sources were 0.03 μM Na_2_SeO_3_ and 0.057 μM (NH_4_)_6_Mo_7_O_24_. pH was altered to 7.5 and maintained with 20 mM HEPES. The final recipe is included in Appendix S1. In addition, all media concentrations used in the media optimization process are found in Table S1.

### 4.2 Culturing and Growth Measurements

For all growth and nutrient optimizations, a starter culture of *C. zofingiensis* SAG 211‐14 was inoculated at ~1 × 10^5^ cells mL^−1^ from agar plates grown in constant light. All test growth curves were diluted from the starter liquid culture to 1 × 10^5^ cells mL^−1^ on inoculation day. Cells were grown in 50 mL of culture in 250 mL plastic beakers with petri dish lids that provided gas exchange inside an Infors HT Multitron Pro growth chamber with 100 μmol photons m^−2^ s^−1^ constant light, 150 rpm shaking, 25°C, and approximately 80% controlled humidity. The appropriate glucose concentration was added from a 2 M stock solution at 4 or 5 days after inoculation.

Cell density and volumetric biomass were assessed daily by a Multisizer 3 (Beckman Coulter). Volumetric biomass was calculated and extracted from Coulter Counter files by an R script described in Jeffers et al. 2024.

### 4.3 Glucose Concentration Measurements

To measure external glucose in the spent medium, a small amount of media supernatant (~0.5 mL) was separated from cells by centrifugation. Samples were either assayed immediately according to the Megazyme D‐Glucose Assay Kit (K‐GLUHKR) or placed in a −20°C freezer. On wet ice, a master mix was prepared fresh containing 50 mM HEPES (pH 7.5), 5 mM MgCl2, 1.33 mM NADP+, and 4 mM ATP. A standard curve of glucose concentrations from 0 to 1 mM was established in technical triplicates, diluted to a final 200 μL volume with master mix in a 96‐well plate. Technical triplicates of each spent medium sample were added to wells and diluted with master mix to be between 0 and 1 mM glucose. Absorbance at 340 nm was measured, and then 2 μL of Megazyme hexokinase ± glucose‐6‐phosphate dehydrogenase (“Reagent 2”) that was stored at 4°C was added to each well, and the change of absorbance in each well was assayed over 20 min using a Tecan Infinite M1000 Pro (Tecan, USA) and used to quantify glucose concentration.

### 4.4 Quantifying Elemental Concentrations by Inductively Coupled Plasma Mass‐Spectrometry

A range of 4–10 mL of culture were transferred to a 50 mL‐falcon tube, which was centrifuged for 3 min at 3220 g in an Eppendorf 5810R centrifuge. A fraction (< 0.5 mL) of the supernatant was saved for spent medium analyses and frozen at −20°C. The pellets were resuspended in ~10 mL of 1 mM Na2EDTA (pH 8.0) solution for three consecutive wash/centrifugation steps to remove residual cell‐associated mineral nutrients. The final wash steps took place in 15 mL falcon tubes and included a final wash with 10 mL of Millipore water to remove remnants of EDTA before the analysis. The cell pellets were frozen at −20°C. All glassware used to hold EDTA solutions was acid washed in 50% HCl as previously described (Glaesener et al. 2013).

Prior to ICP‐MS/MS analysis, cell pellets were digested with 70% ICP‐MS grade nitric acid, first at room temperature overnight, then followed by additional incubation at 65°C until the cell pellet was completely dissolved. Elemental concentrations were quantified for both cell pellets and spent medium in an Agilent 8900 Triple Quadrapole ICP‐MS/MS using methods and standardization as described (Schmollinger et al. 2021). Ionic measurements were either normalized to the volumetric biomass of the pellet or by moles of sulfur.

### 4.5 Lipid Extraction and Thin Layer Chromatography

TLC was conducted as described previously in Roth, Westcott, et al. 2019 and Jeffers et al. 2024. Two milliliters of culture was pelleted by centrifugation (20,000 × g, 3 min). Lipids were extracted by resuspending pellets in 1 mL chloroform: methanol (with 0.01% BHT), followed by bead beating in Lysing Matrix D tubes with the FastPrep‐24 5G High‐Speed Homogenizer (3 × 60 s, 6.5 m/s, MP Biomedicals). After adding 266 μL of 0.73% NaCl, samples were centrifuged (20,000 × g, 5 min), and the lower organic phase was transferred to glass tubes and evaporated. Lipids were resuspended in 100 μL hexane, and equal volumes (10 μL) of extracted lipid were applied to the TLC plate to allow for inferences into total culture bioproductivity of TAGs. One milliliter of olive oil was used as TAG standard. Plates were run with solvent hexane: diethyl ether: glacial acetic acid (91:39:1.3), stained with 0.05% Primuline (for UV detection). The TLC plate was imaged using UV transmission using the Bio‐Rad ChemiDoc MP Imaging System. The photograph of the TLC plate was inverted to the complementary image in Figure 5B in Adobe Photoshop.

### 4.6 FAMEs Quantification

Two milliliters of culture were pelleted by centrifugation (20,000 × g, 3 min) and lipids were extracted as described for TLC. An internal standard of 12.5 μg of tritridecanoin (a C13:0 triacylglycerol) was added to each sample. One milliliter of 1 N methanolic HCl was used to perform acid‐catalyzed transmethylation to form FAMEs. Tubes were sealed with PTFE‐lined caps and incubated at 80°C for 30 min. After cooling, 1 mL of 0.9% NaCl and 1 mL hexane were added, and samples were vortexed and centrifuged (3000 × g, 5 min). The upper hexane phase (~1.5 mL) containing FAMEs was transferred, dried under N_2_, and resuspended in 1 mL hexane. A total of 20 μL of the FAME‐containing hexane phase was injected into a gas chromatograph with flame ionization detection (GC‐FID with high‐resolution gas chromatography, Agilent Technologies 7890A GC system equipped with a 7683B series injector and an Agilent J&W gas chromatography column G3903‐63012; 0.250 mm ID, 0.25 μm film thickness). Data acquisition and analysis were carried out using Agilent GC OpenLab software. Fatty acid peaks were detected based on shared retention times with reference to a 37‐component FAME standard mixture (Agilent). Each identified fatty acid per sample was quantified based on its peak area in relation to the peak of the tritridecanoin standard. The total FAME content in each sample is determined as the sum of all identified FAME peaks.

### 4.7 Pigments Identification

Pigments were determined using high‐performance liquid chromatography (HPLC; 1100 HPLC Agilent) as previously described by Roth et al. 2017 and Roth, Gallaher, et al. 2019, with slight modifications. Two milliliters of culture were pelleted by centrifugation (20,000 × g, 3 min) and homogenized with 400 μL 100% (v/v) ethanol and lysing matrix D with the FastPrep‐24 5G High‐Speed Homogenizer (3 × 60 s, 6.5 m/s, MP Biomedicals). The homogenate was cooled briefly on ice after each cycle. The ethanol extraction was repeated three times. The homogenate was centrifuged (20,000 × g, 3 min) and the 0.22 μm filtered supernatant was run on the HPLC system. The HPLC system was equipped with a YMC Carotenoid C30 Reversed‐Phase Column (250 × 4.6 mm I.D., S‐5 μm particle size, column volume 4.2 mL; YMC America), as described by Gupta et al. (2015). Pigments were detected at 445 nm with reference at 550 nm. Absorbance spectra at 445 nm and standards when available were used to verify pigments.

### 4.8 Statistical Analysis

Welch's two‐tailed *t‐*tests were conducted for volumetric biomass doubling times of +Glc vs. No Glc in ADJ medium (Figure 1B), elemental content (atoms per volumetric biomass) of No Glc vs. +Glc cells (Figure 1E) and comparisons of CORE and Proteose volumetric biomass and FAME quantification in +Glc and No Glc (Figure 5). One‐way ANOVA was conducted for comparisons of growth parameters for derived media (e.g., Figure 2), pH treatments, buffer comparisons, and K/NA ratios, with post hoc Tukey's HSD conducted to identify treatment comparisons that were significantly different based on adjusted *p*‐value. Linear models were conducted for the regression of internal elemental composition as a function of the external levels of that same element. All *t‐*tests, ANOVAs, and linear models were conducted in R (v.4.1.1). Plots were produced with ggplot2. Summary statistics are reported in the Results as mean ± SE.

## Author Contributions

T.L.J., K.K.N., and M.S.R. conceptualized the research. T.L.J., S.S., S.S.M., K.K.N., and M.S.R. designed the research. T.L.J., R.M., S.S., and S.T. performed the research. T.L.J., S.S., S.T., S.U., S.S.M., K.K.N., and M.S.R. analyzed the data. T.L.J. and M.S.R. wrote the paper. All authors contributed to editing and approved the paper.

## Conflicts of Interest

The authors declare no conflicts of interest.

## Peer Review

The peer review history for this article is available in the Supporting Information for this article.

## Supporting information


**Data S1:** Peer review.

**Figure S1:** Increasing nitrogen with glucose improves visible chlorophyll production. An example of cultures in a 24‐well plate resuspended across a nitrate gradient (columns) and glucose gradient (rows). The photograph is taken 4 days after a seed culture was centrifuged and resuspended in the various *N* media with varied Glc concentration in each well. Cultures are grown in Bristols Medium (Bold 1949) with Hutner's micronutrient supplement (Hutner et al. 1950) in 16 h light:8 h dark cycles at 100 μmol photons m^‐2^ s^‐1^. Glucose was only treated once in the cultures.
**Figure S2:** Long‐term glucose‐fed *C. zofingiensis* cultures become high‐density cell slurries. (A) Change in mean cell diameter of +Glc vs. autotrophic cells over time, matching biomass and cell density growth curves of Figure 1A,B. (B) Photographs of ~45 mL + Glc cultures centrifuged after 17 days and 24 days continued maintenance of +Glc and mineral nutrients through resupply (*n* = 2). % cellular volume was approximated from the wet cellular pelleted volume.
**Figure S3:** Manganese and magnesium hyperaccumulated in low nutrient medium. (A) Cell density growth curve (corresponding to biomass growth curve in Figure 2B). (B) Percentage of magnesium (Mg) and manganese (Mn) left in spent medium on day 8 of +Glc growth of various media (x‐axis). Depletion of Mn in P14 was already apparent by day 4. (C) Corresponding elemental hyperaccumulation of Mn in cells on day 7 for both photoautotrophic and +Glc samples in P14. Values are expressed as means ± SE (*n* = 3).
**Figure S4:** pH and buffer impacts on culture growth. pH gradient volumetric biomass growth curves of two experimental replicates, conducted in M10 medium with only phosphorus buffer. For (A), the y‐axis is in log scale while for the same data the y‐axis is not scaled in (B). Colors refer to the pH of media at the start of the experiment. Volumetric biomass (C) and cell density (D) growth curves of cultures with solely phosphate as a buffer (control, red), or with the addition of 20 mM HEPES (purple) or 20 mM Tris (gold). pH 7.5 (solid) and 8 (dashed) were also tested for each buffer treatment and were indistinguishable. (E) Day 7 measurements of volumetric biomass (top), cell density (lower left) and cell diameter (lower right). Statistics is done by pairwise t‐test (N.S. refers to corrected p > 0.05 while ** refers to p < 0.01). (F) pH of media over time of three buffer treatments with media having a starting pH of 7.5. Values are expressed as means ± SE (*n* = 3–6).
**Figure S5:** Effect of nitrogen species and concentration on *C. zofingiensis* growth. (A) Volumetric biomass and (B) cell density growth curves of cultures inoculated into different levels of ammonium and nitrate. Day 6 was not measured in ammonium cultures that failed to grow. (C) Photograph of autotrophic cultures of each treatment on day 3. Besides nitrogen, all other medium parameters pertain to M10 + 20 mM HEPES solution (Table S1). Values are expressed as means ± SE (*n* = 2–3).
**Figure S6:** Effect of potassium to sodium ratio on *C. zofingiensis* growth. (A) Volumetric biomass and (B) cell density growth curves of cultures where potassium to sodium concentration ratios (K/Na) are intentionally altered while the anions NO_3_^–1^ and PO_4_^–3^ are maintained at the same concentrations. “1:1.6 Ratio (CORE)” treatment indicates the CORE medium K/Na, which is the result of using only sodium nitrate and potassium‐phosphate as salt sources in the medium. Beside K and Na, all other medium parameters pertain to M10 medium (Table S1). Values are expressed as means ± SE (*n* = 2–3).
**Figure S7:** No difference in cell density between 50 vs. 200 μM iron medium. Photoautotrophic cell density growth curve of M10 HEPES N Boost medium (Table S1) with 200 μM Fe (gray, triangle, dotted line) vs. 50 μM Fe (beige, circles, solid line). Days 3, 4, and 5 samples were collected for ionomic quantification and are overlaid with red, orange, and green, respectively. The 50 μM Fe medium represents the finalized CORE medium from this study. Values are expressed as means ± SE (*n* = 2–3). Volumetric biomass of the same experiment is found in Figure 4A in the main manuscript.
**Figure S8:** Additional growth parameters of Proteose vs. CORE comparison. (A) Cell Density and (B) mean cell diameter of CORE (beige) and Proteose (light blue) cultures in +Glc (triangle) and No Glc (circles), 96 h after +Glc treatment. Values are expressed as means ± SE (*n *= 3–4).
**Figure S9:** Detection of astaxanthin in CORE vs. Proteose. (A) Photograph of pellet from 2 mL of culture of CORE and Proteose in +Glc and No Glc showing pigmentation differences. (B) HPLC chromatogram from CORE and Proteose in No Glc (green) or +Glc (orange). Pigment abbreviations: vio: violaxanthin, neo: neoxanthin, anth: antheraxanthin, chl *a*: chlorophyll *a*, lut: lutein, chl *b*: chlorophyll *b*, ast: astaxanthin, β‐car: β‐carotene. Pigments were detected at 44 nm with reference at 550 nm. (C) Detail of chromatogram containing esterified astaxanthin peaks. In CORE, peaks at ~21.5 and ~23 min represent mixed pigments of astaxanthin with another unidentified carotenoid. Each line of the chromatogram represents individual extract replicates (*n* = 3–4).

**Appendix S1:** Recipe for finalized CORE medium.

**Table S1:** All media compositions used in the study through optimization.

**Table S2:** Statistical reporting of elemental content differences between No Glc vs. +Glc cells.

**Table S3:** Statistical reporting of linear models showing internal concentration as a function of external concentration per element.

## Data Availability

All relevant data for the manuscript and its Supporting Information can be found in the Open Science Framework (link: https://osf.io/5bjcf/).
