# Supplementary material for: An Algal Nutrient‐Replete, Optimized Medium for Fast Growth and High Triacylglycerol Accumulation
Source: Plant Direct. 2025 Sep 12;9(9):e70106. doi: 10.1002/pld3.70106 (PMC12426764; doi:10.1002/pld3.70106)
Supplement: Supplementary file 2 — Figure S1: Increasing nitrogen with glucose improves visible chlorophyll production. An example of cultures in a 24‐well plate resuspended across a nitrate gradient (columns) and glucose gradient (rows). The photograph is taken 4 days after a seed culture was centrifuged and resuspended in the various N media with varied Glc concentration in each well. Cultures are grown in Bristols Medium (Bold 1949) with Hutner's micronutrient supplement (Hutner et al. 1950) in 16 h light:8 h dark cycles at 100 μmol photons m‐2 s‐1. Glucose was only treated once in the cultures. Figure S2: Long‐term glucose‐fed C. zofingiensis cultures become high‐density cell slurries. (A) Change in mean cell diameter of +Glc vs. autotrophic cells over time, matching biomass and cell density growth curves of Figure 1A,B. (B) Photographs of ~45 mL + Glc cultures centrifuged after 17 days and 24 days continued maintenance of +Glc and mineral nutrients through resupply (n = 2). % cellular volume was approximated from the wet cellular pelleted volume. Figure S3: Manganese and magnesium hyperaccumulated in low nutrient medium. (A) Cell density growth curve (corresponding to biomass growth curve in Figure 2B). (B) Percentage of magnesium (Mg) and manganese (Mn) left in spent medium on day 8 of +Glc growth of various media (x‐axis). Depletion of Mn in P14 was already apparent by day 4. (C) Corresponding elemental hyperaccumulation of Mn in cells on day 7 for both photoautotrophic and +Glc samples in P14. Values are expressed as means ± SE (n = 3). Figure S4: pH and buffer impacts on culture growth. pH gradient volumetric biomass growth curves of two experimental replicates, conducted in M10 medium with only phosphorus buffer. For (A), the y‐axis is in log scale while for the same data the y‐axis is not scaled in (B). Colors refer to the pH of media at the start of the experiment. Volumetric biomass (C) and cell density (D) growth curves of cultures with solely phosphate as a buffer (control, red), or wi [file PLD3-9-e70106-s003.pdf]

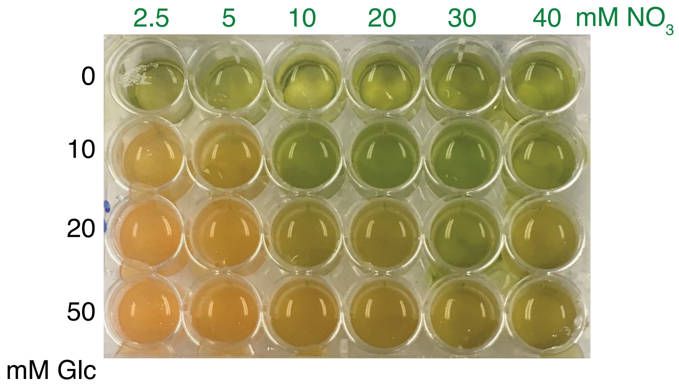

Figure S1. Increasing nitrogen with glucose improves visible chlorophyll production.
An example of cultures in a 24 well-plate resuspended across nitrate gradient (columns) and glucose gradient (rows). Photograph is taken 4 days after a seed culture was centrifuged and resuspended in the various N media with varied Glc concentration in each well. Cultures are grown in Bristols Medium (Bold 1949) with Hutner's micronutrient supplement (Hutner et al., 1950) in 16 hour light:8 hour dark cycles at 100 µmol photons m^{-2} s^{-1}. Glucose was only treated once in the cultures.

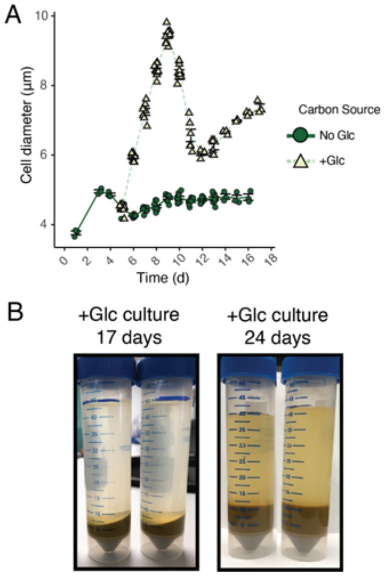

Figure S2. Long-term glucose-fed C. zofingiensis cultures become high-density cell slurries. **(A)** Change in mean cell diameter of +Glc vs autotrophic cells over time, matching biomass and cell density growth curves of Figure 1A,B. **(B)** Photographs of ~45mL +Glc cultures centrifuged after 17 days and 24 days continued maintenance of +Glc and mineral nutrients through resupply (*n* = 2). % cellular volume was approximated from the wet cellular pelleted volume.

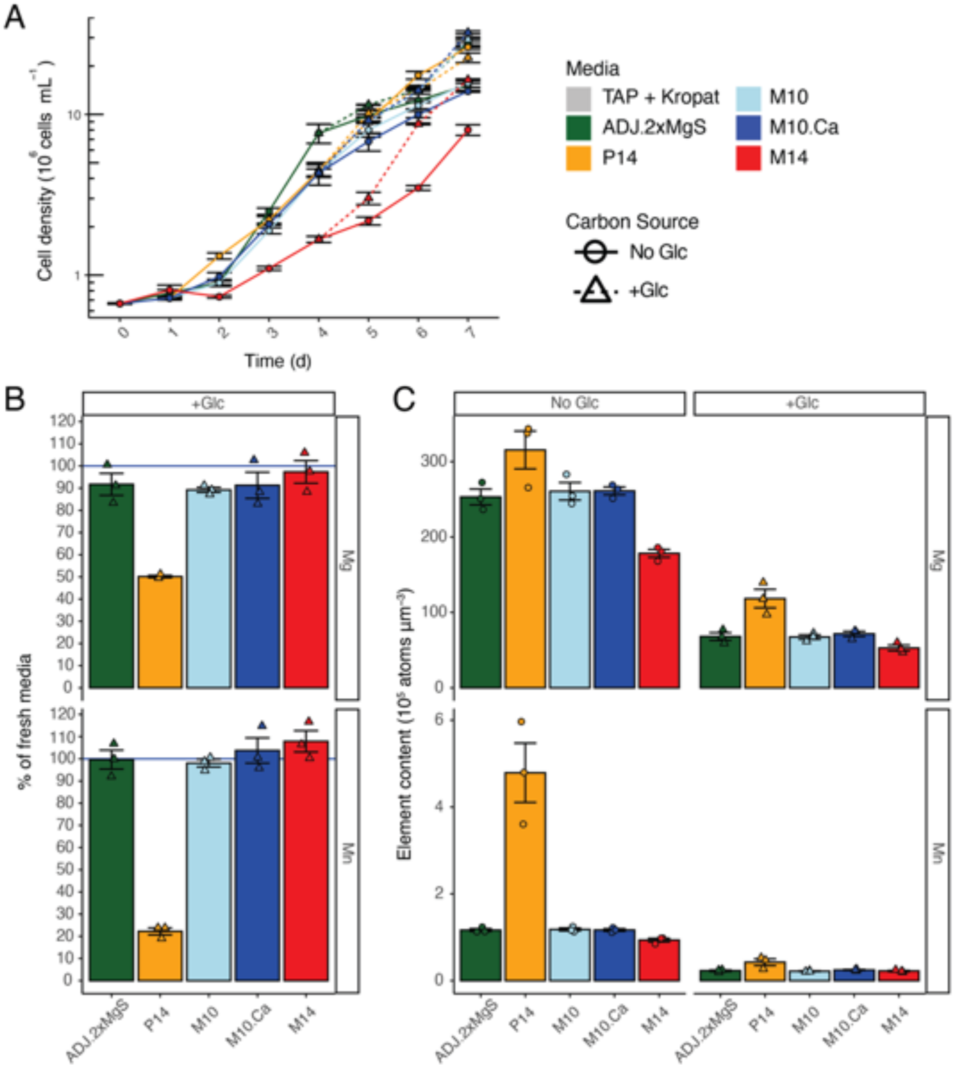

Figure S3. Manganese and magnesium hyperaccumulated in low nutrient medium.

(A) Cell density growth curve (corresponding to biomass growth curve in Figure 2B). **(B)** Percentage of magnesium (Mg) and manganese (Mn) left in spent medium on day 8 of +Glc growth of various media (*x*-axis). Depletion of Mn in P14 was already apparent by day 4. **(C)** Corresponding elemental hyperaccumulation of Mn in cells on day 7 for both photoautotrophic and +Glc samples in P14. Values are expressed as means ± SE (n = 3).

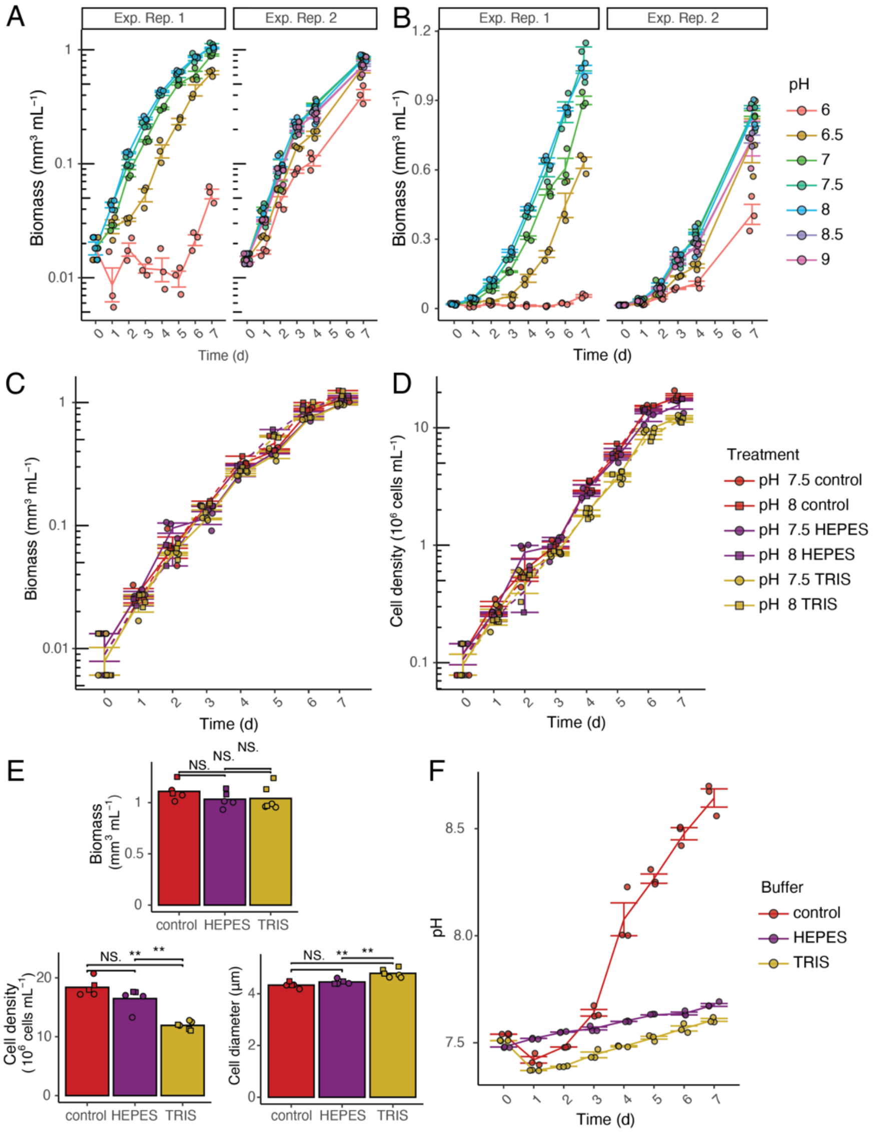

Figure S4. pH and buffer impacts on culture growth.

pH gradient volumetric biomass growth curves of two experimental replicates, conducted in M10 medium with only phosphorus buffer. For **(A)**, *y*-axis is in log scale while for the same data *y*-axis is not scaled in **(B)**. Colors refer to pH of media at the start of the experiment. Volumetric biomass **(C)** and cell density (**D)** growth curves of cultures with solely phosphate as a buffer (control, red), or with the addition of 20 mM HEPES (purple) or 20 mM Tris (gold). pH 7.5 (solid) and 8 (dashed) were also tested for each buffer treatment and were indistinguishable. **(E)** Day 7 measurements of volumetric biomass (top), cell density (lower left) and cell diameter (lower right). Statistics is done by pairwise *t*-test (N.S. refers to corrected $p \geq 0.05$ while ** refers to $p < 0.01$). **(F)** pH of media over time of three buffer treatments with media having a starting pH of 7.5. Values are expressed as means ± SE (n = 3-6).

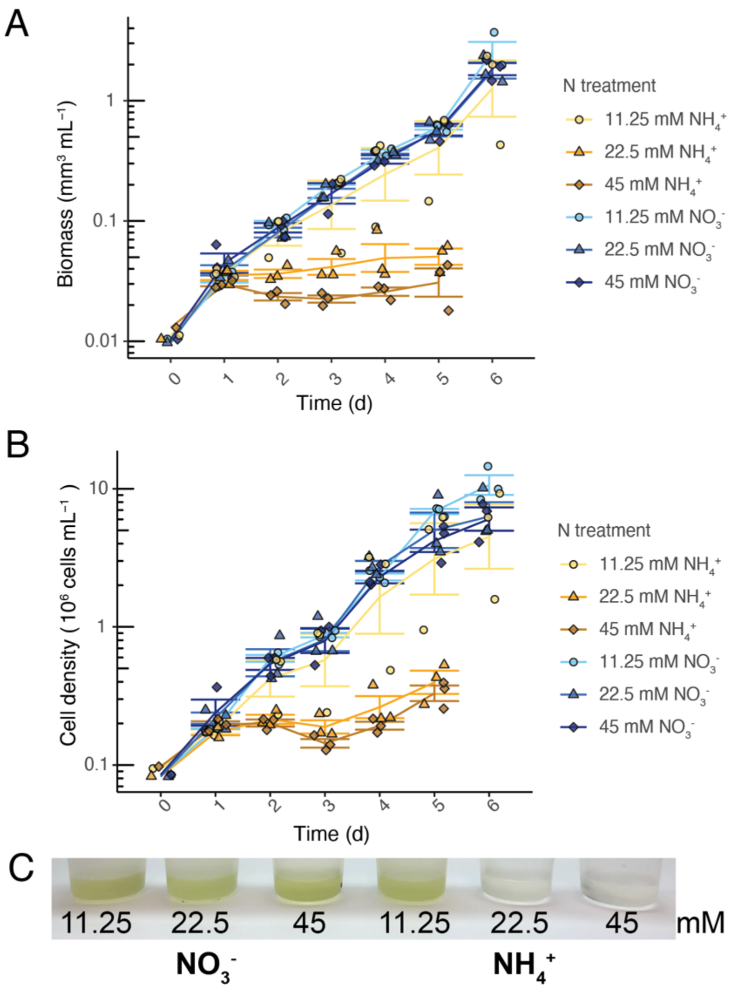

Figure S5. Effect of nitrogen species and concentration on *C. zofingiensis* growth.

(A) Volumetric biomass and **(B)** cell density growth curves of cultures inoculated into different levels of ammonium and nitrate. Day 6 was not measured in ammonium cultures that failed to grow. **(C)** Photograph of autotrophic cultures of each treatment on day 3. Besides nitrogen, all other medium parameters pertain to M10 + 20mM HEPES solution (Table S1). Values are expressed as means $\pm$ SE (n = 2-3).

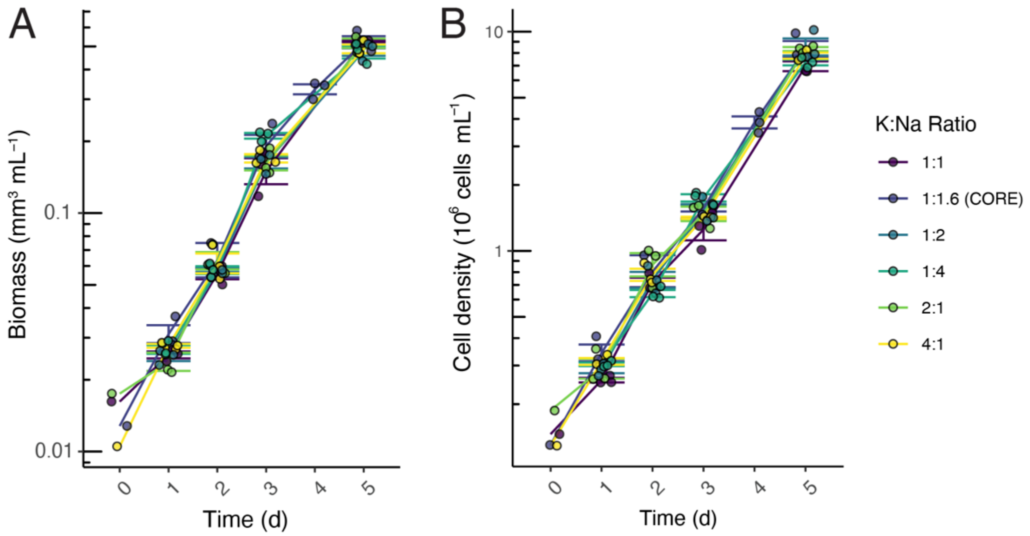

Figure S6. Effect of potassium to sodium ratio on *C. zofingiensis* growth.
(**A)** Volumetric biomass and **(B)** cell density growth curves of cultures where potassium to sodium concentration ratios (K/Na) are intentionally altered while the anions NO_3^{-1} and PO_4^{-3} are maintained at the same concentrations. "1:1.6 Ratio (CORE)" treatment indicates the CORE medium K/Na, which is the result of using only sodium nitrate and potassium-phosphate as salt sources in the medium. Beside K and Na, all other medium parameters pertain to M10 medium (Table S1). Values are expressed as means ± SE (*n* = 2-3).

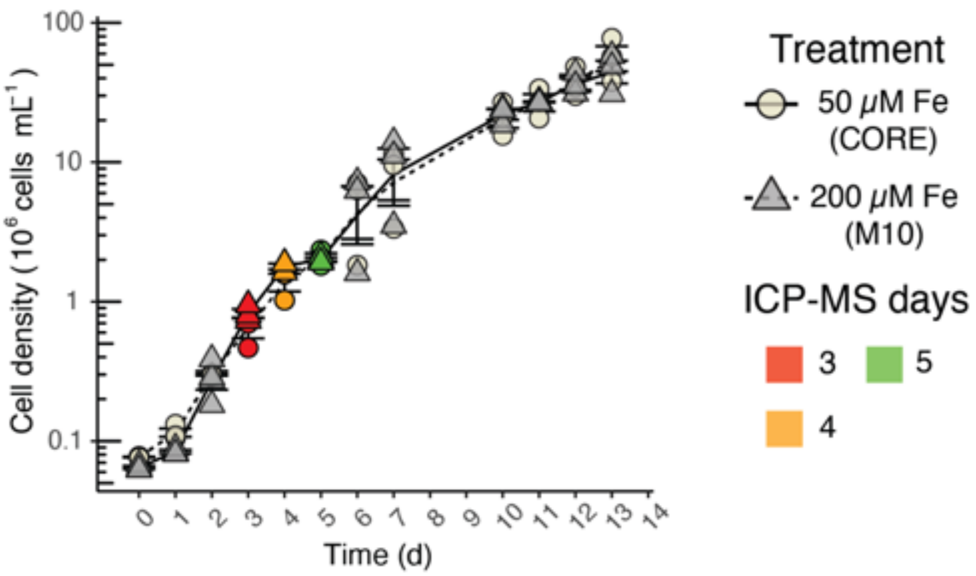

Figure S7. No difference in cell density between 50 vs. 200 µM iron medium.

Photoautotrophic cell density growth curve of M10 HEPES N Boost medium (Table S1) with 200 µM Fe (grey, triangle, dotted line) vs. 50 µM Fe (beige, circles, solid line). Days 3, 4, and 5 samples were collected for ionomic quantification and are overlayed with red, orange, and green, respectively. The 50 µM Fe medium represents the finalized CORE medium from this study. Values are expressed as means ± SE (*n* = 2-3). Volumetric biomass of same experiment is found in Figure 4A in main manuscript.

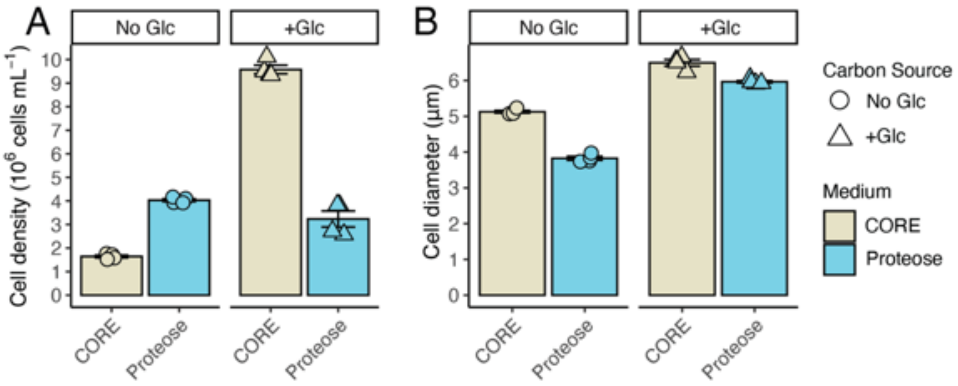

Figure S8. Additional growth parameters of Proteose vs. CORE comparison. (A) Cell Density and **(B)** mean cell diameter of CORE (beige) and Proteose (light blue) cultures in +Glc (triangle) and No Glc (circles), 96 h after +Glc treatment. Values are expressed as means ± SE (*n* = 3-4).

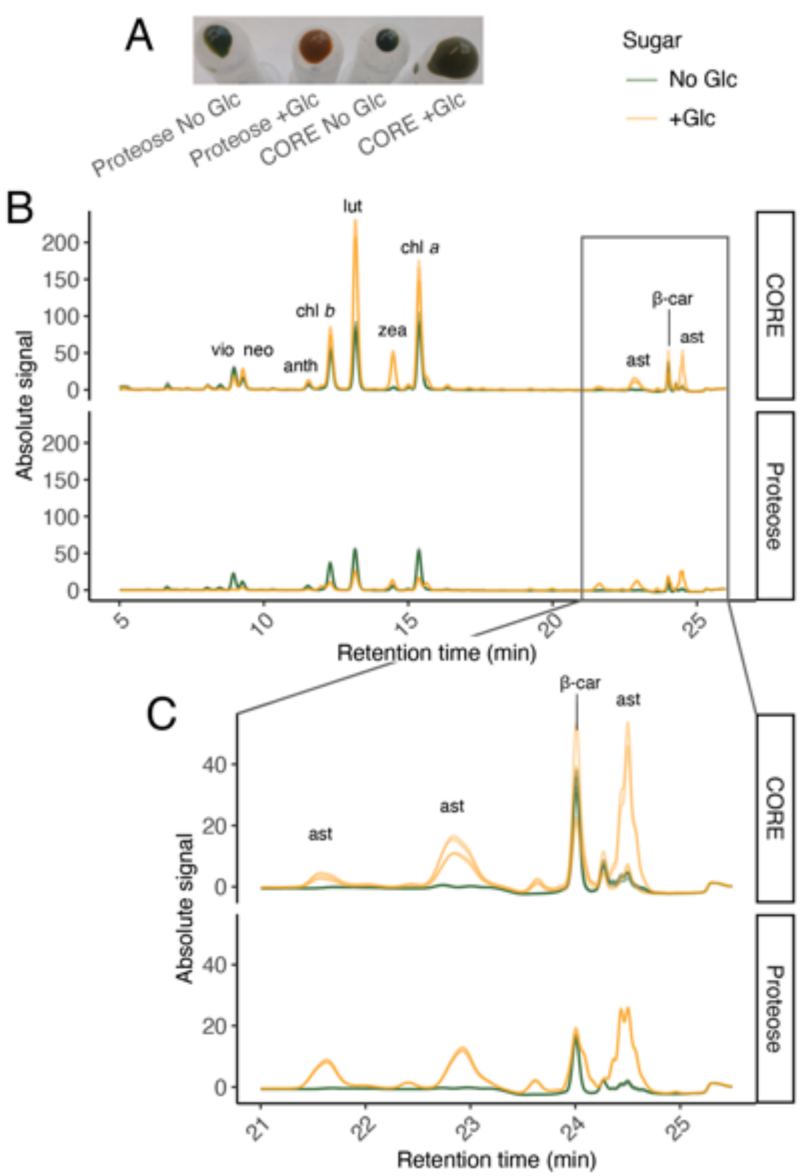

Figure S9. Detection of astaxanthin in CORE vs. Proteose. (A) Photograph of pellet from 2 mL of culture of CORE and Proteose in +Glc and No Glc showing pigmentation differences. **(B)** HPLC chromatogram from CORE and Proteose in No Glc (green) or +Glc (orange). Pigment abbreviations: vio: violaxanthin, neo: neoxanthin, anth: antheraxanthin, chl *a*: chlorophyll *a*, lut: lutein, chl *b*: chlorophyll *b,* ast: astaxanthin, β-car: β-carotene. Pigments were detected at 44 nm with reference at 550 nm. **(C)** Detail of chromatogram containing esterified astaxanthin peaks. In CORE, peaks at ~21.5 min and ~23 min represent mixed pigments of astaxanthin with another unidentified carotenoid. Each line of the chromatogram represents individual extract replicates (*n* = 3-4).

Supplemental References

Bold, H.C. (1949) The Morphology of *Chlamydomonas chlamydogama*, Sp. Nov. *Bulletin of the Torrey Botanical Club*, 76(2).

Hutner, S.H., Provasoli, L., Schatz, A., & Haskins, C.P. (1950). Some approaches to the study of the role of metals in the metabolism of microorganisms. *Proceedings of the American Philosophical Society*, 94, 152-170.
