# Supplementary material for: An Algal Nutrient‐Replete, Optimized Medium for Fast Growth and High Triacylglycerol Accumulation
Source: Plant Direct. 2025 Sep 12;9(9):e70106. doi: 10.1002/pld3.70106 (PMC12426764; doi:10.1002/pld3.70106)
Supplement: Supplementary file 1 — Data S1: Peer review. [file PLD3-9-e70106-s002.pdf]

Plant Direct
Open Access

An algal nutrient-replete, optimized medium for fast growth and high triacylglycerol accumulation

Dr. Tim L Jeffers, Mr. Ryan McCombs , Dr. Stefan Schmollinger , Dr. Srikanth Tirumani , Shivani Upadhyaya , Prof. Sabeeha S Merchant , Krishna K. Niyogi , Dr. Melissa S Roth

Decision Letter Round 1:

January 26, 2025

Dr. Melissa S Roth

University of California Berkeley

Berkeley, California

RE: An algal nutrient-replete, optimized medium for fast growth and high triacylglycerol accumulation

Dear Dr. Roth:

Thank you for submitting to Plant Direct. All required reviews have been returned and we have now finished our evaluation of your manuscript. In light of the reviewers' and editor's comments, further revisions are needed before the paper can be accepted for publication in Plant Direct.

Please view the editors' and reviewers' comments below and use their suggestions as a guide while you work on your revision.

When uploading the revised version of this article, please be sure to include the following:

-A word document that contains your response to the reviewers. You should respond to each reviewer comment and note the changes made to the manuscript. If you do not agree with a reviewer's comment and choose not to make a suggested revision, please explain why. Please try to provide as complete an answer as possible to each reviewer's criticisms.

-A tracked changes document with each change highlighted

- A clean version of the latest version of the manuscript

Comments from Academic Editor:

After careful review, we feel that the submitted manuscript does not fully meet Plant Direct's publication criteria. We invite you to submit a revised manuscript that carefully addresses the points outlined by the reviewers, and this will be considered for potential publication.

Important points to address include:

1. Address similar comments made by Reviewers #2 and #3 regarding the units used to report biomass.

2. To be consistent with the title of the manuscript, the manuscript should include triacylglycerol accumulation data for the strains grow in the optimized medium. The current title of the manuscript extends beyond the results that are presented, i.e. data showing "high triacylglycerol accumulation" is not included (see comment by Reviewer #3).

3. Carefully consider Reviewer 3's comment that the data reported in this manuscript should also be compared to other green algae that utilize glucose (they suggest Chlorella).

4. In the Results section, be careful to present specific results that include quantities and specific details, in addition the descriptive narrative.

To upload your revision, please click the link below.

https://plantdirect.msubmit.net/cgi-bin/main.plex?el=A7Lr6lt6A4pEQ1I5A9ftdc5XS4iOYhKyRsas5baaQAZ

In order to provide as timely a service as possible, we ask that your revision is resubmitted within three months after receipt of this request. If an extension is needed, please send a request, along with a brief explanation, to the editorial office at plantdirect@wiley.com .

Please note that, in addition to publishing reviewer comments, the author's responses to review comments will also be published alongside the final version of the paper. If you would not like the author's responses to be published, please contact the editorial office at plantdirect@wiley.com .

Thank you very much for giving us an opportunity to review your work. I look forward to receiving the next version.

Sincerely,

Marna Yandeau-Nelson

Gustavo MacIntosh

Editor, Plant Direct

-- Editor comments:

After careful review, we feel that the submitted manuscript does not fully meet Plant Direct's publication criteria. We invite you to submit a revised manuscript that carefully addresses the points outlined by the reviewers, and this will be considered for potential publication.

Important points to address include:

1. Address similar comments made by Reviewers #2 and #3 regarding the units used to report biomass.

2. To be consistent with the title of the manuscript, the manuscript should include triacylglycerol accumulation data for the strains grow in the optimized medium. The current title of the manuscript extends beyond the results that are presented, i.e. data showing "high triacylglycerol accumulation" is not included (see comment by Reviewer #3).

3. Carefully consider Reviewer 3's comment that the data reported in this manuscript should also be compared to other green algae that utilize glucose (they suggest Chlorella).

4. In the Results section, be careful to present specific results that include quantities and specific details, in addition the descriptive narrative.

--- Reviewer comments:

Reviewer #1:

The authors have done a careful study of nutrient requirements for growth to produce a baseline media that is optimized for the growth of Chromochloris zofingiensis

In biofuel relevant conditions. Overall, the methods are clear, the experiments are done carefully and with appropriate controls and replication and the conclusions are supported by the data.

The only point I think that the authors should address is the use of the word "intracellular ionome". As far as I can tell, the authors are just measuring the ionome, which we defined in Salt et al. (2008 https://www.annualreviews.org/content/journals/10.1146/annurev.arplant.59.032607.092942) as "the mineral nutrient and trace element composition of an organism". They aren't doing anything to isolate the intracellular compartments of the cell, instead, they are measuring the entire organism, including things bound tightly to the cell wall, plasma membrane and anything in between them. If I am missing something and the authors mean something else by intracellular ionome, they should define it more clearly.

Minor point: I had trouble following the sentence at the top of page 3 "Recently, we discovered that switching off photosynthesis, but not lipid accumulation, is prevented by treating +Glc cultures with a replete iron supplement (Jeffers et al., 2024)." Is the phenotype that is 'prevented' photosynthesis, lipid accumulation or both? It's not clear from reading that section and clarification would be useful.

Reviewer #2:

In this manuscript, Jeffers et al. describe an improve medium for enhanced growth and lipid production in green algae. The motivation is clear and the writing is clear in general. Below are my suggestions.

Because the paper's nature, it's important to put the catalog numbers in the supplemental table 1 by the chemicals. And also include the grams for 1 L of medium besides the final concentration.

In the title of the first section, considering that glucose can also be considered a nutrient, the authors can change "demands more nutrients" into "demands more mineral nutrients."

While it is OK to provide volumetric biomass, for the most critical experiments, it will be helpful to provide cell number or dry weight as well because many people do not have an idea about the volumetric biomass numbers.

It's helpful to readers if the ADJ medium is explained and clearly stated as the base medium for optimization in the first section of Results, rather than in the Methods.

The authors should also give suggestions on whether this medium revision should be used for long-term algal maintenance on agar plates, or recommend against this.

Reviewer #3:

The manuscript by Jeffers et al. describes the design of a customised cultivation medium for the microalgae C. zofingiensis. Starting from established media components (Chu's micronutrients or Bristol's macronutrients), the authors tested the maximum growth capacity of the alga in mixotrophic/heterotrophic cultivation conditions (addition of glucose/resuspension in new media over a period of 24 days). In addition, a fine-tuned nutrient supply and a suitable pH buffer system were established by measuring the intracellular ion balance. This work and the results presented are interesting and important for the developing community of C. zofingiensis but I see some points that need to be improved by the authors.

Major issues:

- An important component of the work is mixotrophic cultivation using glucose. I do not understand why the authors refer their work to the green alga C. reinhardtii (despite being an model organism, it can only metabolise acetate). It would make more sense to compare the growth behaviour to other (green) algae capable of using glucose, e.g. Chlorella. Alternatively, H. lacustris could also serve as a reference for the native production of astaxanthin. Please include those comparisons.

- The results section contains elements of the discussion section and is widely a descriptive narrative. As an example, on page 6 the authors state "The highest nutrient medium M14 had decreased photoautotrophic growth" and "was growth-inhibiting". These statements are not further explained or quantified with details or measured values. In general, the results section would benefit from clear comparisons of designed media with the previous set up by stating measured values for final biomass, obtained growth rate, etc.

- In line with the previous bullet point: The choice of language is too colloquial, and the description of the results is imprecise in some places. Figures are labelled with "Photoautrophic" and "Glucose", which is are suitable labels for Eppendorf tubes in the lab but need to be more scientific for a publication. Please review throughout the entire manuscript.

- The authors state that C. zofingiensis is a biotechnological valuable host for production of astaxanthin and TAGs but failed to demonstrate the impact of the newly designed medium for this purpose in their work. I strongly suggest, the authors include the quantification of astaxanthin and TAG accumulation at suitable time points and compare to previously reports.

- The authors state found, that HEPES was more suitable to maintain an appropriate pH compared to phosphate buffer. Would an increase in phosphate concentrations make sense to compensate the reduced buffering capacity? What are the cost considerations for these two options. Please include such a discussion in the manuscript.

- Biomass accumulation is given in difficult to understand units (10^9 μm^3 per mL). The authors should provide data in the commonly used units gram biomass per litre, cells per mL (as given in Figure S3D) or optical density, at least in addition to the existing data to allow interpretation.

Minor issues:

- Abstract: glucose consumption is abbreviated by "+Glc", however +Glc was frequently for other purposes. Please review

- Include a brief description of "ionome measurements" in the introduction section as this is not a common topic for readers. Where was it applied previously? What are (dis-)advantages of this system? Give reference.

- First paragraph of results section is too general and appears as part of introduction section.

- The measurement of biomass per volume using a Falcon tube on page 5 and Figure 1B is not very scientific. Please provide more suitable values as gram biomass per litre or similar values.

- Please give clear statement that this media is designed for batch cultivations.

- Figure 1E: x-axis needs superscript letters

Decision Letter Round 2:

July 15, 2025

Dr. Tim L Jeffers

University of California Berkeleyy

Berkeley, California

RE: An algal nutrient-replete, optimized medium for fast growth and high triacylglycerol accumulation

Dear Dr. Jeffers:

Thank you for submitting to Plant Direct. All required reviews have been returned and we have now finished our evaluation of your manuscript. In light of the reviewers' and editor's comments, further revisions are needed before the paper can be accepted for publication in Plant Direct.

Plant Direct
Open Access

Please view the editors' and reviewers' comments below and use their suggestions as a guide while you work on your revision.

When uploading the revised version of this article, please be sure to include the following:

-A word document that contains your response to the reviewers. You should respond to each reviewer comment and note the changes made to the manuscript. If you do not agree with a reviewer's comment and choose not to make a suggested revision, please explain why. Please try to provide as complete an answer as possible to each reviewer's criticisms.

-A tracked changes document with each change highlighted

- A clean version of the latest version of the manuscript

Comments from Academic Editor:

After careful review of this revised manuscript, it is evident that the authors have addressed the concerns and suggestions provided by the reviewers, in part by adding new experiments and additional analysis. However, we feel that the submitted manuscript requires an additional modification to meet Plant Direct's publishing criteria. Please address Reviewer #3's concern regarding the the results presented in Figure S4, and the interpretation of these results for choice of Tris versus HEPES, which could be done by modifying the text or by presenting additional data. We invite you to submit a revised manuscript that addresses this point.

To upload your revision, please click the link below.

https://plantdirect.msubmit.net/cgi-bin/main.plex?el=A4Lr2lt2B4pDs5I5A9ftdYzWo4z7i8cpq0PizfUrjwgZ

In order to provide as timely a service as possible, we ask that your revision is resubmitted within three months after receipt of this request. If an extension is needed, please send a request, along with a brief explanation, to the editorial office at plantdirect@wiley.com .

Please note that, in addition to publishing reviewer comments, the author's responses to review comments will also be published alongside the final version of the paper. If you would not like the author's responses to be published, please contact the editorial office at plantdirect@wiley.com .

Thank you very much for giving us an opportunity to review your work. I look forward to receiving the next version.

Sincerely,

Marna Yandeau-Nelson

Gustavo MacIntosh

Editor, Plant Direct

-- Editor comments:

After careful review of this revised manuscript, it is evident that the authors have addressed the concerns and suggestions provided by the reviewers, in part by adding new experiments and additional analysis. However, we feel that the submitted manuscript requires an additional modification to meet Plant Direct's publishing criteria. Please address Reviewer #3's concern regarding the the results presented in Figure S4, and the interpretation of these results for choice of Tris versus HEPES, which could be done by modifying the text or by presenting additional data. We invite you to submit a revised manuscript that addresses this point.

-- Reviewer comments:

Reviewer #2:

The authors have fully addressed my comments. I endorse the publication of this manuscript.

Reviewer #3:

Thank you very much for carefully correcting the manuscript. The changes you have made have significantly improved this publication, which will be a valuable resource for the microalgae community. However, I have one remaining concern:

The authors state that "[...],Tris was found to suppress cell division." (page 11) and likely refer to data presented in Suppl. Figure S4.

In addition, the authors state on page 5: "To incorporate the cell size data, we report the cumulative volumetric sum (µm3) per mL of cells as a more accurate description of biomass than the more commonly used cell density, which is consistent with previous publications (Roth et al., 2019a; Roth et al. 2019b; Jeffers et al. 2024)"

However, a close look at Suppl. Figure S4 part E reveals, that values on biomass accumulation given in µm3 per mL are not significantly different in HEPES or TRIS conditions, which can be misleading and is at least in part a contradiction to the previous statement.

The authors should expand their statement on page 11 that the biomass accumulation was not significantly altered, only the measured cell count was reduced and thus revise the justification for the use of HEPES in the discussion section as indicated in the results section.

Alternatively, they can revise their statement on the presentation of biomass accumulation on page 5 and present data on cell number and cell diameter separately throughout the manuscript, which would provide more clarity for all the data presented. This would also be in line with the authors' statement that the representation of the cell number is more commonly used in the microalgae community.

Decision Letter Round 3:

August 4, 2025

Dr. Tim L Jeffers

University of California Berkeleyy

Plant Direct
Open Access

Berkeley, California

MSID: 2024-01677R2

MS TITLE: An algal nutrient-replete, optimized medium for fast growth and high triacylglycerol accumulation

Dear Dr. Tim L Jeffers:

I am pleased to inform you that your manuscript "An algal nutrient-replete, optimized medium for fast growth and high triacylglycerol accumulation" has been accepted for publication in Plant Direct.

Your article will appear online in the next available issue of Plant Direct. To ensure your article gets published as quickly as possible, please pay attention to the steps detailed below. We have found that most of the delays happen at this stage, especially at the payment stage, so please respond as quickly as possible when prompted.

License Agreement: Once your article has been accepted it will move to Production and undergo admin and file checking - you may receive an email with any queries we have at this stage. When all required items are received by the publisher and queries resolved, the corresponding author will receive an email from Wiley's Author Services system which will ask them to log on at https://authorservices.wiley.com/bauthor and will present them with the appropriate license for completion. Your article cannot be published until both the signed license agreement and payment of the article fee have been received.

Payment of the Open Access Article Publication Fee: All articles published in Plant Direct are fully open access: immediately and freely available to read, download and share. Plant Direct charges a publication fee to cover the publication costs. The corresponding author for this manuscript should have already received a quote from the payments team (cs-openaccess@wiley.com) with the estimated article publication fee; please email cs-openaccess@wiley.com if this has not been received. The corresponding author should log on to the Wiley Author Services site, where the publication fee can be paid by credit card or an invoice. Pro Forma can also be requested. Payment of the publication charge must be received before the article will be published online.

Proofs: You will have the opportunity to look over your paper once more when you receive the author proofs for your article. The proofs will be with you in approximately two weeks. Please note that, in addition to publishing reviewer comments, the author's responses to review comments will also be published alongside the final version of the paper. If you would not like the author's responses to be published, please contact the editorial office at plantdirect@wiley.com .

Promotion of your article: You can help your research get the attention it deserves! Check out Wiley's free Promotion Guide for best-practice recommendations for promoting your work at www.wileyauthors.com/eeo/guide. And learn more about Wiley Editing Services which offers professional video, design, and writing services to create shareable video abstracts, infographics,

conference posters, lay summaries, and research news stories for your research at www.wileyauthors.com/eeo/promotion.

Thank you again for your contribution to Plant Direct. If you have any questions, feel free to contact the editorial office at plantdirect@wiley.com .

Sincerely,

Marna Yandeau-Nelson

Gustavo MacIntosh

Editor, Plant Direct

-- Editor comments

The revised version of the manuscript addresses reviewer suggestions. We are pleased to accept this manuscript for publication.

-- Reviewer comments:

Response Letter Round 1:

After careful review, we feel that the submitted manuscript does not fully meet Plant Direct's publication criteria. We invite you to submit a revised manuscript that carefully addresses the points outlined by the reviewers, and this will be considered for potential publication.

We are pleased to be invited to submit a revised manuscript. We have carefully addressed the points outlined by the reviewers in the manuscript and in our responses to the editor and reviewers. We thank the editor and reviewers for their comments and believe that our manuscript has been strengthened in the revision.

Important points to address include:

1. Address similar comments made by Reviewers #2 and #3 regarding the units used to report biomass.

Thank you for your point. We have revised our manuscript to report cell density as well as volumetric biomass (cell density data in Figure 1B; Figure S3A; Figure S5B; Figure S6; Figure S7; Figure S8A).

2. To be consistent with the title of the manuscript, the manuscript should include

triacylglycerol accumulation data for the strains grow in the optimized medium.
The current title of the manuscript extends beyond the results that are presented, i.e. data showing "high triacylglycerol accumulation" is not included (see comment by Reviewer #3).
Thank you for this point. We have added an experiment comparing the optimized medium and the original medium with biomass (and cell density measurements), TAG measurements by TLC, total FAME and TAG-enriched FAME signatures quantification by GC-FID, and HPLC of pigments to address this point and Reviewer #3's comment. We indeed show improved growth and lipid productivity in the optimized medium compared to our formerly used medium in +Glc conditions (Figure 5, Figure S8, Figure S9). These measurements show the improvement of the medium for a green alga that can concurrently increase biomass and storage lipid productivity, an important physiological trait with powerful implications for biotechnology.
3. Carefully consider Reviewer 3's comment that the data reported in this manuscript should also be compared to other green algae that utilize glucose (they suggest Chlorella).
Thank you for this point. We have added in comparisons with other green algae including Chlorella.
4. In the Results section, be careful to present specific results that include quantities and specific details, in addition the descriptive narrative.
Thank you for this point. Throughout the manuscript, we have added measured values and statistical tests in addition to the descriptive narrative in the Results section.
--- Reviewer comments:
Reviewer #1:
The authors have done a careful study of nutrient requirements for growth to produce a baseline media that is optimized for the growth of Chromochloris zofingiensis
In biofuel relevant conditions. Overall, the methods are clear, the experiments are done carefully and with appropriate controls and replication and the conclusions

are supported by the data.

Thank you for the positive description of our study and manuscript. Thank you for your suggestions as they have improved our manuscript.

The only point I think that the authors should address is the use of the word "intracellular ionome". As far as I can tell, the authors are just measuring the ionome, which we defined in Salt et al. (2008 https://www.annualreviews.org/content/journals/10.1146/annurev.arplant.59.032607.092942) as "the mineral nutrient and trace element composition of an organism". They aren't doing anything to isolate the intracellular compartments of the cell, instead, they are measuring the entire organism, including things bound tightly to the cell wall, plasma membrane and anything in between them. If I am missing something and the authors mean something else by intracellular ionome, they should define it more clearly.

Thank you for the thoughtful comments. We changed the text to clarify that the ionome refers to the ion composition of cellular biomass. We also changed the term “internal concentration” of elements to “cellular concentration” of elements.

Minor point: I had trouble following the sentence at the top of page 3 "Recently, we discovered that switching off photosynthesis, but not lipid accumulation, is prevented by treating +Glc cultures with a replete iron supplement (Jeffers et al., 2024)." Is the phenotype that is 'prevented' photosynthesis, lipid accumulation or both? It's not clear from reading that section and clarification would be useful.

Thank you, this sentence has been rewritten for clarity.

Reviewer #2:

In this manuscript, Jeffers et al. describe an improve medium for enhanced growth and lipid production in green algae. The motivation is clear and the writing is clear in general. Below are my suggestions.

Thank you for the positive comments about our study and manuscript. Thank you for your suggestions as they have improved our manuscript.

Because the paper's nature, it's important to put the catalog numbers in the

supplemental table 1 by the chemicals. And also include the grams for 1 L of medium besides the final concentration.

Thank you for the suggestion. We have added both g L-1

and catalog numbers

information to Table S1.

In the title of the first section, considering that glucose can also be considered a nutrient, the authors can change "demands more nutrients" into "demands more mineral nutrients."

Thank you, we have specified mineral nutrients in the title of the first section and throughout the manuscript.

While it is OK to provide volumetric biomass, for the most critical experiments, it will be helpful to provide cell number or dry weight as well because many people do not have an idea about the volumetric biomass numbers.

Thank you for pointing this out. We have added cell density data for every volumetric biomass measurement (Figure 1B, Figure S3A; Figure S5B; Figure S6; Figure S7; Figure S8A).

It's helpful to readers if the ADJ medium is explained and clearly stated as the base medium for optimization in the first section of Results, rather than in the Methods.

Thank you, we have added the statement that ADJ is the base medium for optimization at the last sentence of the second paragraph in Results section 1.

The authors should also give suggestions on whether this medium revision should be used for long-term algal maintenance on agar plates, or recommend against this.

Thank you, this is a great point. We indeed use CORE to effectively maintain long-term growth on agar plates. We added this flexibility of use of this media in the Discussion.

Reviewer #3:

The manuscript by Jeffers et al. describes the design of a customised cultivation medium for the microalgae C. zofingiensis. Starting from established media

components (Chu's micronutrients or Bristol's macronutrients), the authors tested the maximum growth capacity of the alga in mixotrophic/heterotrophic cultivation conditions (addition of glucose/resuspension in new media over a period of 24 days). In addition, a fine-tuned nutrient supply and a suitable pH buffer system were established by measuring the intracellular ion balance. This work and the results presented are interesting and important for the developing community of C. zofingiensis but I see some points that need to be improved by the authors.

Thank you for your positive review of our manuscript and study. We appreciate that you found the work and results "interesting and important for the developing community". We thank you for points to improve the study and believe that it has strengthened our manuscript.

Major issues:

- An important component of the work is mixotrophic cultivation using glucose. I do not understand why the authors refer their work to the green alga C. reinhardtii (despite being an model organism, it can only metabolise acetate). It would make more sense to compare the growth behaviour to other (green) algae capable of using glucose, e.g. Chlorella. Alternatively, H. lacustris could also serve as a reference for the native production of astaxanthin. Please include those comparisons.

Thank you for the comment. We have added to the introduction descriptions of glucose's influence on growth and TAG accumulation in other algae (other Sphaeropleales, Chlorella and Auxenochlorella, Introduction, third paragraph). Additionally, we have added statements on the astaxanthin accumulation in Haematococcus lacustris (Introduction, second paragraph). We also added a statement and reference to a study comparing TAG accumulation of 96 species including C. zofingiensis (Introduction, second paragraph).

Unfortunately Chlorella, Auxenochlorella, and Haematococcus etc. lack data on their cellular ionome, and thus, we compare our ionomics data to the published ionomic data

of another Chlorophyceae, C. reinhardtii, despite its distinct metabolism to C. zofingiensis. However, we noted the differences between species, describe the caveats of this comparison, and clarify the unique considerations for C. zofingiensis.

- The results section contains elements of the discussion section and is widely a descriptive narrative. As an example, on page 6 the authors state "The highest nutrient medium M14 had decreased photoautotrophic growth" and "was growthinhibiting". These statements are not further explained or quantified with details or measured values. In general, the results section would benefit from clear comparisons of designed media with the previous set up by stating measured values for final biomass, obtained growth rate, etc.

Thank you for these points. We have added measured values and statistical tests for these specific points and throughout the Results. We have also added a clear comparison of the designed media with the previous medium through an additional experiment. We have added the Results section “CORE Medium improves lipid productivity through enhanced biomass” and the associated Figure 5, Figure S8, and Figure S9 and describe the differences in biomass and cell density.

- In line with the previous bullet point: The choice of language is too colloquial, and the description of the results is imprecise in some places. Figures are labelled with "Photoautrophic" and "Glucose", which is are suitable labels for Eppendorf tubes in the lab but need to be more scientific for a publication. Please review throughout the entire manuscript.

Thank you for this suggestion. We have updated the treatment descriptions to “No Glc” and +Glc, which more precisely describes the treatment and was used in our previous publications (Roth et al., 2019a; Roth et al. 2019b).

- The authors state that C. zofingiensis is a biotechnological valuable host for production of astaxanthin and TAGs but failed to demonstrate the impact of the newly designed medium for this purpose in their work. I strongly suggest, the authors include the quantification of astaxanthin and TAG accumulation at suitable time points and compare to previously reports.

Thank you for this comment. We have added an experiment comparing our original medium and our optimized medium and believe this strengthened our manuscript. We added the Results section "CORE Medium improves lipid productivity through enhanced biomass" and the associated Figure 5 and Figure S8 that show how quantifications of biomass and lipid productivity by TLC and GC-FID CORE medium improves lipid productivity in +Glc conditions. In Figure S9, we also incorporate HPLC chromatograms of C. zofingiensis pigments to show that astaxanthin is also increased in +Glc in CORE.

- The authors state found, that HEPES was more suitable to maintain an appropriate pH compared to phosphate buffer. Would an increase in phosphate concentrations make sense to compensate the reduced buffering capacity? What are the cost considerations for these two options. Please include such a discussion in the manuscript.

Thank you for this question. We added a commentary on this to the manuscript in the Discussion section (paragraph 4). We chose not to increase phosphate levels because the current phosphorus concentrations of our medium reflect the same ratio as calculated from the cellular ionome. Higher phosphorus in media increases precipitation with divalent cations like Mg^{2+} and Ca^{2+}. Like other publications (Zepernick et al., 2020) we found HEPES buffer was necessary to maintain pH.

- Biomass accumulation is given in difficult to understand units (10^9 µm³ per mL). The authors should provide data in the commonly used units gram biomass per litre, cells per mL (as given in Figure S3D) or optical density, at least in addition to the existing data to allow interpretation.

Thank you for your comment. We have added cell density data for every volumetric biomass calculation (cell density data in Figure 1B, Figure S3A; Figure S5B; Figure S6; Figure S7; Figure S8A).

Minor issues:

- Abstract: glucose consumption is abbreviated by "+Glc", however +Glc was frequently for other purposes. Please review.

Thank you for pointing that out. We have clarified this by only using +Glc to precisely

indicate glucose treatment.

- Include a brief description of "ionome measurements" in the introduction section as this is not a common topic for readers. Where was it applied previously? What are (dis-)advantages of this system? Give reference.

Thank you for the suggestion. We have provided a definition and background of ionomics in the introduction that clarifies its methodology and role in biology, especially in determining healthy ranges of mineral nutrients in algae.

- First paragraph of results section is too general and appears as part of introduction section.

Thank you for the comment. To address this point, we have moved most of the content of this paragraph to the Introduction.

- The measurement of biomass per volume using a Falcon tube on page 5 and Figure 1B is not very scientific. Please provide more suitable values as gram biomass per litre or similar values.

Thank you for the comment. We replaced the photograph from Figure1B with a graph showing cell density differences of this culture.

- Please give clear statement that this media is designed for batch cultivations.

Thank you for the comment. We have clarified that this media is designed for batch cultivations and expand on the topic in the discussion.

- Figure 1E: x-axis needs superscript letters. Thank you, we corrected Figure 1E.

New References in Response to Editors

Zepernick, B.N., Krausfeldt, L.E. & Wilhelm, S.W. (2020) Flaming as part of aseptic technique increases CO_2(g) and decreases pH in freshwater culture media. Limnology and Oceanography: Methods, 18(5), 211–219

Response Letter Round 2:

-- Editor comments:

After careful review of this revised manuscript, it is evident that the authors have

addressed the concerns and suggestions provided by the reviewers, in part by adding new experiments and additional analysis. However, we feel that the submitted manuscript requires an additional modification to meet Plant Direct's publishing criteria. Please address Reviewer #3's concern regarding the the results presented in Figure S4, and the interpretation of these results for choice of Tris versus HEPES, which could be done by modifying the text or by presenting additional data. We invite you to submit a revised manuscript that addresses this point.

Thank you. The points on buffer use have been clarified in the main manuscript per Reviewer #3's request.

-- Reviewer comments:

Reviewer #2:

The authors have fully addressed my comments. I endorse the publication of this manuscript.

Reviewer #3:

Thank you very much for carefully correcting the manuscript. The changes you have made have significantly improved this publication, which will be a valuable resource for the microalgae community. However, I have one remaining concern:

The authors state that "[...],Tris was found to suppress cell division." (page 11) and likely refer to data presented in Suppl. Figure S4.

In addition, the authors state on page 5: "To incorporate the cell size data, we report the cumulative volumetric sum (µm3) per mL of cells as a more accurate description of biomass than the more commonly used cell density, which is consistent with previous publications (Roth et al., 2019a; Roth et al. 2019b; Jeffers et al. 2024)"

However, a close look at Suppl. Figure S4 part E reveals, that values on biomass accumulation given in µm3 per mL are not significantly different in HEPES or TRIS conditions, which can be misleading and is at least in part a contradiction to the previous statement.

The authors should expand their statement on page 11 that the biomass accumulation was not significantly altered, only the measured cell count was reduced and thus revise

the justification for the use of HEPES in the discussion section as indicated in the results section.

Alternatively, they can revise their statement on the presentation of biomass accumulation on page 5 and present data on cell number and cell diameter separately throughout the manuscript, which would provide more clarity for all the data presented. This would also be in line with the authors' statement that the representation of the cell number is more commonly used in the microalgae community.

We appreciate the comments that have improved this manuscript. We have made changes on page 11 to be explicit that biomass has not changed to clarify Tris only impacted cell division.
